# Frontotemporal lobar degeneration proteinopathies have disparate microscopic patterns of white and grey matter pathology

**DOI:** 10.1186/s40478-021-01129-2

**Published:** 2021-02-23

**Authors:** Lucia A. A. Giannini, Claire Peterson, Daniel Ohm, Sharon X. Xie, Corey T. McMillan, Katya Raskovsky, Lauren Massimo, EunRah Suh, Vivianna M. Van Deerlin, David A. Wolk, John Q. Trojanowski, Edward B. Lee, Murray Grossman, David J. Irwin

**Affiliations:** 1 Digital Neuropathology Laboratory, Department of Neurology, Perelman School of Medicine, University of Pennsylvania, Philadelphia, PA 19104 USA; 2 Department of Neurology, Perelman School of Medicine, Penn Frontotemporal Degeneration Center (FTDC), Hospital of the University of Pennsylvania, 3600 Spruce Street, Philadelphia, PA 19104 USA; 3 Department of Neurology, Alzheimer Center, Erasmus University Medical Center, Rotterdam, The Netherlands; 4 Department of Biostatistics, Epidemiology and Informatics, Perelman School of Medicine, University of Pennsylvania, Philadelphia, PA 19104 USA; 5 Department of Pathology and Laboratory Medicine, Center for Neurodegenerative Disease Research, Perelman School of Medicine, University of Pennsylvania, Philadelphia, PA 19104 USA; 6 Department of Pathology and Laboratory Medicine, Alzheimer’s Disease Center, Perelman School of Medicine, University of Pennsylvania, Philadelphia, PA 19104 USA; 7 Department of Neurology, Perelman School of Medicine, University of Pennsylvania, Philadelphia, PA 19104 USA; 8 Translational Neuropathology Research Laboratory, Department of Pathology and Laboratory Medicine, Perelman School of Medicine, University of Pennsylvania, Philadelphia, PA 19104 USA

**Keywords:** Tau, TDP-43, Frontotemporal dementia, Primary progressive aphasia, Neuropathology

## Abstract

Frontotemporal lobar degeneration proteinopathies with tau inclusions (FTLD-Tau) or TDP-43 inclusions (FTLD-TDP) are associated with clinically similar phenotypes. However, these disparate proteinopathies likely differ in cellular severity and regional distribution of inclusions in white matter (WM) and adjacent grey matter (GM), which have been understudied. We performed a neuropathological study of subcortical WM and adjacent GM in a large autopsy cohort (n = 92; FTLD-Tau = 37, FTLD-TDP = 55) using a validated digital image approach. The *antemortem* clinical phenotype was behavioral-variant frontotemporal dementia (bvFTD) in 23 patients with FTLD-Tau and 42 with FTLD-TDP, and primary progressive aphasia (PPA) in 14 patients with FTLD-Tau and 13 with FTLD-TDP. We used linear mixed-effects models to: (1) compare WM pathology burden between proteinopathies; (2) investigate the relationship between WM pathology burden and WM degeneration using luxol fast blue (LFB) myelin staining; (3) study regional patterns of pathology burden in clinico-pathological groups. WM pathology burden was greater in FTLD-Tau compared to FTLD-TDP across regions (beta = 4.21, SE = 0.34, *p* < 0.001), and correlated with the degree of WM degeneration in both FTLD-Tau (beta = 0.32, SE = 0.10, *p* = 0.002) and FTLD-TDP (beta = 0.40, SE = 0.08, *p* < 0.001). WM degeneration was greater in FTLD-Tau than FTLD-TDP particularly in middle-frontal and anterior cingulate regions (*p* < 0.05). Distinct regional patterns of WM and GM inclusions characterized FTLD-Tau and FTLD-TDP proteinopathies, and associated in part with clinical phenotype. In FTLD-Tau, WM pathology was particularly severe in the dorsolateral frontal cortex in nonfluent-variant PPA, and GM pathology in dorsolateral and paralimbic frontal regions with some variation across tauopathies. Differently, FTLD-TDP had little WM regional variability, but showed severe GM pathology burden in ventromedial prefrontal regions in both bvFTD and PPA. To conclude, FTLD-Tau and FTLD-TDP proteinopathies have distinct severity and regional distribution of WM and GM pathology, which may impact their clinical presentation, with overall greater severity of WM pathology as a distinguishing feature of tauopathies.

## Introduction

Frontotemporal lobar degeneration (FTLD) is a heterogeneous spectrum of age-associated neurodegenerative diseases that are currently classified based on the main protein constituents of intracellular aggregations detectable at autopsy. The two main proteinopathies include tauopathies (FTLD-Tau) and TDP-43 proteinopathies (FTLD-TDP) [38]. FTLD proteinopathies are a common etiology of frontotemporal dementia (FTD) clinical phenotypes, which are frequently diagnosed prior to the age of 65 (i.e. young-onset dementia) [26]. FTD clinical phenotypes with underlying FTLD-Tau and FTLD-TDP are clinically similar and there is no diagnostic marker available to reliably predict the underlying neuropathology *antemortem*. Moreover, there are no current FDA-approved therapies, although emerging therapeutic strategies that target protein-specific mechanisms necessitate accurate *antemortem* diagnosis and differentiation of FTLD-Tau and FTLD-TDP proteinopathy groups [4].

Clinicopathological correlations of *antemortem* FTD clinical phenotypes and *postmortem* FTLD neuropathological diagnoses are complex [26]. While syndromic variants of primary progressive aphasia (PPA) [20] have some predictive value of underlying proteinopathy, the most common clinical phenotype, i.e. behavioral-variant FTD (bvFTD) [59], corresponds to roughly equal frequencies of FTLD-Tau and FTLD-TDP proteinopathies at autopsy. Moreover, clinical criteria of PPA variants remains challenging due to the common overlap of language clinical features, which leaves many patients unclassifiable with poor correlation to underlying neuropathology [17, 18, 44]. *Antemortem* neuroimaging patterns of regional atrophy in living patients with clinical PPA and bvFTD suggest that regional patterns of neurodegeneration in interconnected brain regions comprising functional cognitive networks are influential for clinical symptomology in FTD [64]. Yet, it remains unclear how disparate FTLD proteinopathies cause somewhat similar clinical FTD phenotypes. Despite this major gap in knowledge, there are few autopsy studies directly comparing FTLD-Tau and FTLD-TDP, and most of these were performed prior to the discovery of TDP-43 as the pathological substrate for FTD and amyotrophic lateral sclerosis (ALS) [1, 54], did not account for clinical phenotype or did not include the full spectrum of tauopathies [29, 79]. Moreover, while most work has focused on grey matter (GM) pathology, there is very limited comparative study of white matter (WM) pathology in FTLD. We and others previously found divergent regional patterns of microscopic GM pathology across hemispheres in FTLD-Tau compared to FTLD-TDP with clinical PPA [18, 44], suggesting that these specific proteinopathies have distinct patterns of cellular degeneration, which may influence the regional patterns of disease in cognitive networks to yield somewhat different clinical phenotypes. We also previously reported relatively distinct clinicopathological associations of microscopic GM pathology in FTLD-Tau compared to FTLD-TDP with clinical bvFTD [27]. In small cohorts [27, 41], greater relative WM pathology has been reported in FTLD-Tau compared to clinically similar FTLD-TDP. However, no study thus far has rigorously quantified the severity of WM pathology in a large autopsy cohort of FTLD-Tau and FTLD-TDP, and few have examined whether the differential severity of pathology or the regional anatomic distribution of WM pathology contributes to specific FTD clinical phenotypes [23, 58]. Examination of both WM and GM pathology is critical in clinicopathologic studies given current neurocognitive models of large-scale network degradation in neurodegenerative disease [61].

Here, we report a large-scale digital histopathological study in a well-characterized autopsy cohort of bvFTD and PPA patients to address this knowledge gap and test the following hypotheses: (1) there is greater WM pathologic burden across regions in FTLD-Tau subtypes compared to subtypes of FTLD-TDP; (2) WM pathology burden is related to greater WM degeneration in FTLD-Tau compared to FTLD-TDP; and (3) there are distinct regional patterns of WM and GM pathology in FTLD-Tau and FTLD-TDP proteinopathies and their subtypes, which are in part related to clinical phenotype. These large-scale, parametric autopsy data suggest that neuropathological substrates of FTD clinical phenotypes have somewhat distinct cellular signatures of neurodegeneration implicating both WM and GM pathology, and these observations may help to guide future efforts to model human disease and improve *antemortem* diagnosis of FTLD neuropathology.

## Materials and methods

### Patients

We included patients with primary FTLD pathologies meeting modern clinical criteria for PPA [20] or bvFTD [59]. Patients were evaluated at the Penn Frontotemporal Degeneration Center or Alzheimer’s Disease Center by an experienced cognitive neurologist (MG, DAW, DJI), and autopsies were performed at the Penn Center for Neurodegenerative Disease Research (CNDR). Patient data were retrieved from the Penn Integrated Neurodegenerative Disease Database [77] as of September 2017. Clinical diagnosis of PPA or bvFTD was confirmed based on systematic chart review performed by experienced investigators (CTM, DAW, DJI, KR, LAAG, LM, MG). Clinical features of language, behavior and motor disorders (i.e. parkinsonism, motor neuron disease) were extracted from the clinical charts as previously described [19, 24], and summarized in the Supplementary Methods. Patients with a primary pathologic diagnosis of AD or a moderate-to-severe level of secondary AD co-pathology [47] were excluded. Two patients were excluded because they had atypical tau pathology, with no underlying genetic mutation, and could thus not be classified in any of the recognized FTLD-Tau subtypes. Our final cohort consisted of 92 patients with autopsy-confirmed FTLD-Tau (N = 37) or FTLD-TDP (N = 55). We previously reported clinical and quantitative pathology data for 27 patients with PPA [18] and 23 patients with bvFTD [27] in a subset of regions. All procedures were performed with informed consent according to the Declaration of Helsinki and following the regulations of the Penn Institutional Review Board.

### Neuropathological examination

Fresh tissue was sampled at autopsy in standardized regions for diagnosis and fixed overnight in 10% neutral buffered formalin. Tissue was processed as described [25, 69], embedded in paraffin blocks and cut into 6 µm sections for immunohistochemical staining for tau, Aβ, TDP-43 and alpha-synuclein with well-characterized antibodies [69]. Neuropathological diagnosis was performed by expert neuropathologists (EBL, JQT) using established criteria [35, 37, 38, 47]. Patients were classified based on primary neuropathological diagnosis of FTLD-TDP (i.e. subtypes A, B, C or E) or FTLD-Tau (i.e. corticobasal degeneration [CBD], Pick’s disease [PiD], progressive supranuclear palsy [PSP], or tau associated with *MAPT* gene mutation [MAPT]). In FTLD-Tau, we grouped all hereditary cases as a separate subtype, as the *MAPT* gene has been associated with a distinct, heterogeneous spectrum of morphological inclusions [16].

### Genetic analysis

Patients were genotyped for pathogenic mutations in *GRN*, *C9orf72, MAPT* and other neurodegenerative disease-associated genes based on family history risk from structured pedigree analysis as described [69, 76].

### Immunohistochemistry and digital image analysis

Pathology data included five “core” regions and three “extended” regions as described [18, 27]. Core regions were sampled from a random hemisphere at autopsy according to standardized NIA/AA diagnostic guidelines in the total cohort [47]. These core GM regions and subjacent WM regions are the anterior cingulate gyrus (ACG, Brodmann area [BA] 24), angular gyrus (ANG, BA 39), middle frontal cortex (MFC, BA 46), orbitofrontal cortex (OFC, BA 11), and superior-temporal gyrus (STG, BA 22). Extended GM and WM regions were sampled from both hemispheres in more recent autopsies since 2005 (FTLD-Tau = 16, FTLD-TDP = 14) to capture anatomic substrates associated with language and behavior in FTLD as described [27], i.e. anterior insular cortex (INS, BA 13), ventrolateral temporal cortex (VLT, BA 20), and the superior parietal lobule (SPL, BA 5) as a control region less involved in FTLD.

For this study, we used tissue fixed in formalin in an identical manner. A minority of slides (N = 31/664, 4.7% of total slides) were fixed in 70% ethanol with 150 mmol NaCl to supplement regions missing formalin-fixed tissue as previously validated [25]. Tissue was immunostained for phosphorylated TDP-43 (rat monoclonal TAR5P-1D3, p409/410; Ascenion) [52], tau (AT8; Invitrogen) [43] and adjacent sections in unilateral core regions were stained for myelin using luxol fast blue (LFB) as described [41]. Whole-slide images at 20 × magnification were obtained using a Lamina (Perkin Elmer) slide scanning system as described [18, 27]. Digital image analysis was performed with Halo software v2.0 (Indica Labs, Albuquerque NM) with empirically derived thresholding algorithms for each staining batch for FTLD-Tau and FTLD-TDP as previously validated [19] (Supplementary Table 1).

**Table 1:** Demographic and autopsy features by proteinopathy groups and subtypes in our autopsy cohort

	FTLD-Tau	CBD	MAPT	PiD	PSP	FTLD-TDP	Type A	Type B	Type C	Type E	sig. group^a^	sig. subtype^b^
N	37	11	5	12	9	55	20	17	13	5		
Female (%)	16/37 (43.2)	7/11 (63.6)	4/5 (80.0)	4/12 (33.3)	1/9 (11.1)	27/55 (49.1)	11/20 (55.0)	8/17 (47.1)	4/13 (30.8)	4/5 (80.0)	0.672	0.072
Phenotype (%)												
bvFTD	23/37 (62.2)	4/11 (36.4)	5/5 (100)	10/12 (83.3)	4/9 (44.4)	42/55 (76.4)	14/20 (70.0)	16/17 (94.1)	7/13 (53.8)	5/5 (100.0)	0.166	0.004
PPA	14/37 (37.8)	7/11 (63.6)	0/5 (0)	2/12 (16.7)	5/9 (55.6)	13/55 (23.6)	6/20 (30.0)	1/17 (5.9)	6/13 (46.2)	0/5 (0)		
naPPA	10/14 (71.4)	5/7 (71.4)	–	0/2 (0)	5/5 (100.0)	3/13 (23.1)	2/6 (33.3)	1/1 (100.0)	0/6 (0)	–	0.007	< 0.001
svPPA	1/14 (7.1)	0/7 (0)	–	1/2 (50.0)	0/5 (0)	8/13 (61.5)	2/6 (33.3)	0/1 (0)	0/6 (0)	–		
mPPA	3/14 (21.4)	2/7 (28.6)	–	1/2 (50.0)	0/5 (0)	2/13 (15.4)	2/6 (33.3)	0/1 (0)	6/6 (100.0)	–		
Genetic cases (%)	5/37 (13.5)	0/11 (0)	5/5 (100)	0/11 (0)	0/9 (0)	28/55 (50.9)	14/20 (70.0)^c^	12/17 (70.6)	2/13 (15.4)	0/5 (0.0)	< 0.001	
* MAPT*	5/37 (13.5)^d^	0/11 (0)	5/5 (100)	0/11 (0)	0/9 (0)							
* C9orf72*						15/55 (27.3)	3/20 (15.0)	11/17 (64.7)	1/13 (7.7)	0/5 (0.0)		< 0.001^g^
* GRN*						11/55 (20.0)^e^	10/20 (50.0)	1/17 (5.9)	0/13 (0.0)	0/5 (0.0)		
* TBK1*						2/55 (3.6)^f^	1/20 (5.0)	0/17 (0.0)	1/13 (7.7)	0/5 (0.0)		
Age at onset	60.0 ± 12.3	58.1 ± 9.4^#^	47.0 ± 14.9^#^	56.8 ± 6.8^#^	73.6 ± 7.7	59.0 ± 7.7	60.4 ± 6.4^#^	57.7 ± 8.8^#^	59.2 ± 8.5^#^	57.6 ± 8.4^#^	0.691	< 0.001
Age at death	67.6 ± 12.5	64.4 ± 10.6^#^	53.8 ± 16.0^#^	67.3 ± 8.3	79.6 ± 7	66.0 ± 8.8	67.5 ± 7.1^#^	63.7 ± 9.2^#^	68.9 ± 9.9	60.0 ± 9.1^#^	0.496	< 0.001
Disease duration	7.6 ± 3.7	6.3 ± 2.9	6.8 ± 2.3	10.5 ± 4.2^^&^	6.0 ± 2.5	6.9 ± 4.3	7.1 ± 3.6	6.0 ± 4.8	9.6 ± 3.7^&^	2.4 ± 0.9	0.394	< 0.001
PMI	12.0 ± 6.4	13.9 ± 7.4	9.4 ± 3.1	12.6 ± 6.4	10.6 ± 6.5	12.6 ± 6.6	10.6 ± 5.9	14.4 ± 7.7	13.8 ± 6.0	11.6 ± 6.8	0.689	0.550
Brain weight	1091.9 ± 146.7	1096.0 ± 140.7	1082.0 ± 192.7	1021.5 ± 126.5	1179.0 ± 123.3	1097.7 ± 190.3	985.9 ± 165.1^&^	1145.0 ± 207.3	1126.0 ± 134.1	1289.0 ± 119.7	0.872	0.004

bvFTD = behavioral variant of frontotemporal dementia; CBD = corticobasal degeneration; FTLD-Tau = frontotemporal lobar degeneration with inclusions of the protein tau; FTLD-TDP = frontotemporal lobar degeneration with inclusions of the TDP-43 protein; MAPT = FTLD-tau with *MAPT* gene mutation; mPPA = mixed variant of primary progressive aphasia; naPPA = nonfluent variant of primary progressive aphasia; PiD = Pick’s disease; PMI = post-mortem interval; PPA = primary progressive aphasia; PSP = progressive supranuclear palsy; svPPA = semantic variant of primary progressive aphasia; Type A/Type B/Type C/Type E = subtypes of FTLD-TDP

^a^Statistical significance (*p *value) when comparing the two main FTLD proteinopathies (i.e. FTLD-Tau and FTLD-TDP)

^b^Statistical significance (*p *value) when comparing all proteinopathy subtypes (i.e. FTLD-Tau: CBD, MAPT, PiD, PSP; FTLD-TDP: type A, type B, type C, type E)

^c^One patient with FTLD-TDP type A had two VUS in the *GRN* gene (GRN c.956 T > A, p.Ile319Lys; c.1058G > A, p.Ser353Asn), which were not considered to be pathogenic; therefore, this patient was classified as a sporadic case. Another patient with FTLD-TDP type A had a mutation in the *GBE* gene (GBE1 c.1280delG, p.G427fs*9), whose association with FTLD-TDP is unclear; for this reason, this patient was also classified as a sporadic case

^d^*MAPT* point mutations were: c.1165G > A, p.G389R (n = 1); c.796C > G, p.L266V (n = 1); c.902C > T, p.P301L (n = 1); c.915 + 16C > T, intronic variant (n = 2)

^e^*GRN* point mutations were: c.1009C > T, p.Q337* (n = 1 type A); c.102delC, p.G35Efs*19 (n = 1 type A); c.1179 + 2 T > C, p.? (n = 1 type B); c.1252C > T, p.R418* (n = 1 type A); c.1414-2A > G, p.A472Vfs*10 (n = 1 type A); c.1477C > T, p.R493* (n = 1 type A); c.26C > A, p.A9D (n = 1 type A); c.295_308delTGCCCACGGGGCTT, p.C99Pfs*15 (n = 1 type A); c.348A > C, p.S116 = (n = 1 type A); c.675_676delCA, p.S226Wfs*28 (n = 1 type A); c.911G > A, p.W304* (n = 1 type A)

^f^*TBK1* point mutations were: c.1387_1388delGA, p.E463Sfs*13 (n = 1 type C); TBK1 c.922C > T, p.R308* (n = 1 type A)

^g^The frequency of FTLD-TDP-related genetic mutations (i.e. *C9orf72*, *GRN, TBK1*) was compared between proteinopathy subtypes of FTLD-TDP only (i.e. type A, type B, type C, type E). The frequency of FTLD-Tau-related genetic mutations (i.e. *MAPT*) could not be statistically tested between subtypes because all cases with mutation were grouped as a separate MAPT proteinopathy subtype

^#^< 0.05 compared to PSP (*post-hoc* comparisons, Bonferroni-corrected); ^< 0.05 compared to TDP type B (*post-hoc* comparisons, Bonferroni-corrected); ^&^< 0.01 compared to TDP type E (*post-hoc* comparisons, Bonferroni-corrected)

We measured the burden of pathology as the percentage of area occupied (%AO) by TDP-43 or tau positive-pixels in WM and GM regions of interest (ROI) as described [18, 25, 27]. Pathology burden scores were validated by comparison to traditional ordinal ratings (i.e. 0–3), obtained blinded to quantitative pathology measurements [19] (Supplementary Fig. 1). GM ROIs were obtained using a transect-belt sampling method as the longest stretch of parallel cortex to avoid bias from overrepresentation of cortical layers [2]. WM ROIs were obtained as the deepest available WM (i.e. below the sulcal depths) in each cortical tissue section. The mean from a random sample of 175 µm tiles for each GM and WM ROI in each image was used to generate the %AO measurement, as described previously [25]. Our total dataset consisted of 1284%AO measurements from 92 patients, of which 638 were in GM and 646 in WM. Missing data and damaged tissue were excluded from the analyses. We provide an overview of all available %AO measurements per region and pathology group in Supplementary Table 2.

**Fig. 1:**
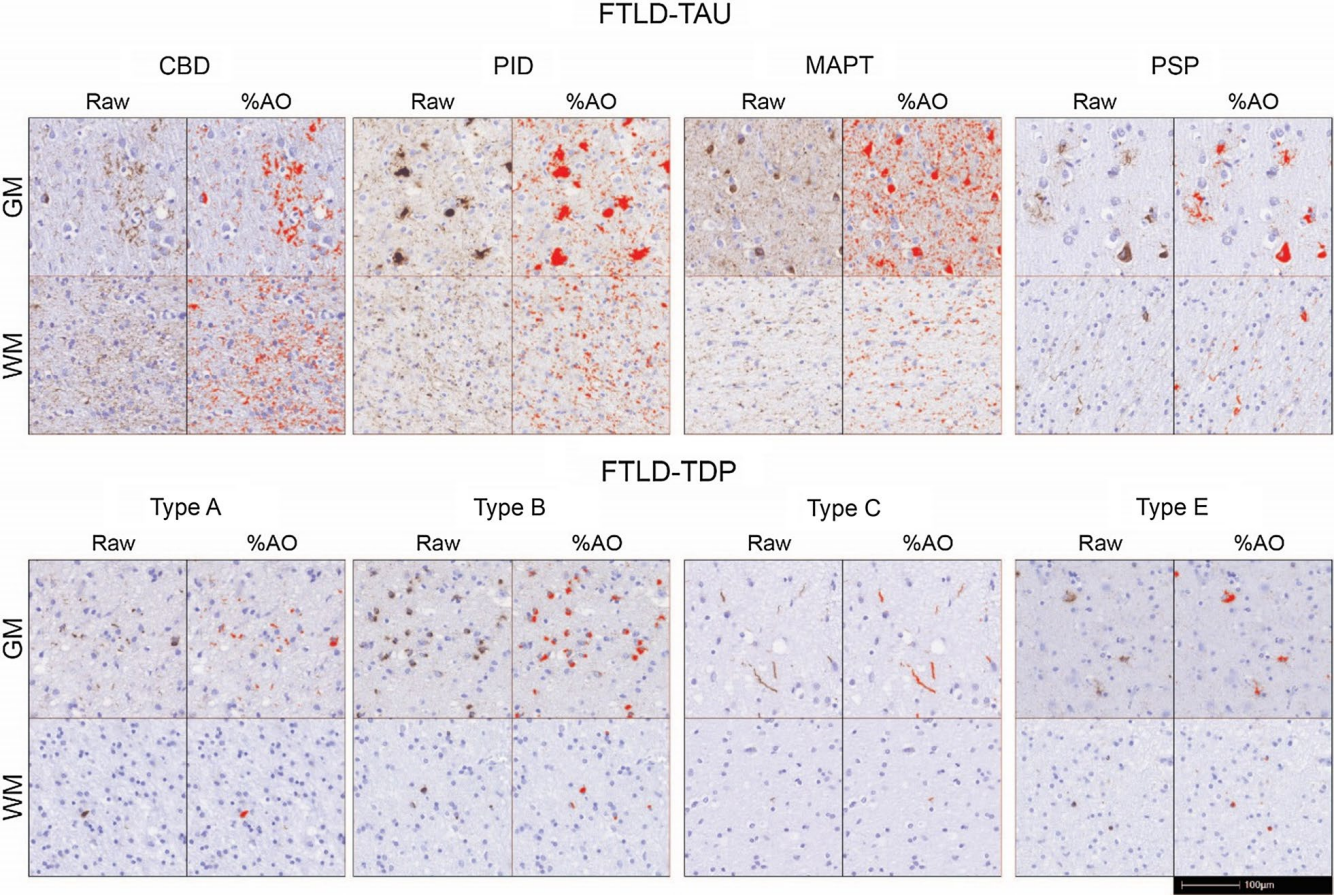
WM and GM pathology burden in FTLD-Tau and FTLD-TDP subtypes. Raw pathology photomicrographs and red digital overlay of %AO detection in middle frontal cortex of each proteinopathy subtype: all FTLD-Tau subtypes display abundant white matter pathology in glia and axonal threads, whereas in FTLD-TDP subtypes white matter pathology are less prominent and largely restricted to oligodendrocytes. Scale bar = 100 µm. Legend: %AO = percentage area occupied by pathology; CBD = corticobasal degeneration; FTLD-Tau = frontotemporal lobar degeneration with inclusions of the tau protein; FTLD-TDP = frontotemporal lobar degeneration with inclusions of the TDP-43 protein; GM = grey matter; MAPT = tau with *MAPT* gene mutation; PiD = Pick’s disease; PSP = progressive supranuclear palsy; Type A/Type B/Type C/Type E = subtypes of FTLD-TDP pathology; WM = white matter

**Table 2:** Clinical features at baseline and follow-up by pathology group and subtype

	FTLD-Tau	CBD	MAPT	PiD	PSP	FTLD-TDP	Type A	Type B	Type C	Type E
Tot. clinical data (N cases)	36	11	5	11	9	53	19	16	13	5
Onset to first visit, years^a^	3 (2–4)	1 (1–4)	4 (4–6)	3 (3–4)	3 (2–3)	2 (1–4)	2 (1–3)	2.5 (2–4)	3 (2–5)	2 (0–2)
N visits^a^	5 (2–10)	7 (3–11)	2 (1–2)	8 (4–10)	4 (2–10)	3 (2–5)	5 (2–9)	2 (1–3)	2 (2–5)	3 (2–4)
BASELINE(0–3 years)^b^										
Available data, N (%)	24/36 (66.7)	8/11 (72.7)	1/5 (20)	8/11 (72.7)	7/9 (77.8)	37/53 (69.8)	15/19 (78.9)	10/16 (62.5)	7/13 (53.8)	5/5 (100)
Behavioral features, N (%)										
Apathy/Inertia	13/24 (54.2)	3/8 (37.5)	1/1 (100)	5/8 (62.5)	4/7 (57.1)	29/37 (78.4)	14/15 (93.3)	9/10 (90)	2/7 (28.6)	4/5 (80)
Disinhibition	19/24 (79.2)	5/8 (62.5)	1/1 (100)	8/8 (100)	5/7 (71.4)	29/37 (78.4)	12/15 (80)	8/10 (80)	4/7 (57.1)	5/5 (100)
Empathy	12/24 (50)	4/8 (50)	0/1 (0)	5/8 (62.5)	3/7 (42.9)	16/37 (43.2)	6/15 (40)	3/10 (30)	2/7 (28.6)	5/5 (100)
Hyperoral behavior	13/24 (54.2)	2/8 (25)	1/1 (100)	7/8 (87.5)	3/7 (42.9)	19/37 (51.4)	8/15 (53.3)	5/10 (50)	2/7 (28.6)	4/5 (80)
Ritualistic behavior	14/24 (58.3)	5/8 (62.5)	0/1 (0)	7/8 (87.5)	2/7 (28.6)	21/37 (56.8)	7/15 (46.7)	8/10 (80)	3/7 (42.9)	3/5 (60)
Language features, N (%)										
Word-finding diff	16/24 (66.7)	8/8 (100)	0/1 (0)	3/8 (37.5)	5/7 (71.4)	27/37 (73)	11/15 (73.3)	8/10 (80)	6/7 (85.7)	2/5 (40)
Agrammatism	8/24 (33.3)	6/8 (75)	0/1 (0)	1/8 (12.5)	1/7 (14.3)	9/37 (24.3)	4/15 (26.7)	3/10 (30)	1/7 (14.3)	1/5 (20)
Imp gramm compr	7/24 (29.2)	6/8 (75.0)	0/1 (0)	0/8 (0)	1/7 (14.3)	11/37 (29.7)	6/15 (40)	3/10 (30)	2/7 (28.6)	0/5 (0)
Imp naming	11/24 (45.8)	6/8 (75)	0/1 (0)	3/8 (37.5)	2/7 (28.6)	19/37 (51.4)	10/15 (66.7)	4/10 (40)	5/7 (71.4)	0/5 (0)
Nonfluent speech	15/24 (62.5)	7/8 (87.5)	1/1 (100)	2/8 (25)	5/7 (71.4)	14/37 (37.8)	9/15 9 (60)	3/10 (30)	1/7 (14.3)	1/5 (20)
Imp repetition	4/24 (16.7)	3/8 (37.5)	0/1 (0)	1/8 (12.5)	0/7 (0)	8/37 (21.6)	4/15 (26.7)	3/10 (30)	0/7 (0)	1/5 (20)
Imp single-word compr	3/24 (12.5)	2/8 (25)	0/1 (0)	1/8 (12.5)	0/7 (0)	10/37 (27)	4/15 (26.7)	1/10 (10)	4/7 (57.1)	1/5 (20)
Other features, N (%)										
MND signs	0/24 (0)	0/8 (0)	0/1 (0)	0/8 (0)	0/7 (0)	6/37 (16.2)	1/15 (6.7)	4/10 (40)	0/7 (0)	1/5 (20)
Parkinsonism	7/24 (29.2)	2/8 (25)	0/1 (0)	1/8 (12.5)	4/7 (57.1)	18/37 (48.6)	11/15 (73.3)	2/10 (20)	1/7 (14.3)	4/5 (80)
FOLLOW-UP (> 3 years)^c^										
Available data, N (%)	27/36 (75.0)	8/11 (72.7)	4/5 (80)	9/11 (81.8)	6/9 (66.7)	31/53 (58.5)	11/19 (57.9)	8/16 (50)	11/13 (84.6)	1/5 (20)
Behavioral features, N (%)										
Apathy/Inertia	24/27 (88.9)	6/8 (75)	3/4 (75)	9/9 (100)	6/6 (100)	27/31 (87.1)	10/11 (90.9)	8/8 (100)	8/11 (72.7)	1/1 (100)
Disinhibition	24/27 (88.9)	7/8 (87.5)	4/4 (100)	9/9 (100)	4/6 (66.7)	26/31 (83.9)	9/11 (81.8)	7/8 (87.5)	9/11 (81.8)	1/1 (100)
Empathy	17/27 (63)	5/8 (62.5)	2/4 (50)	7/9 (77.8)	3/6 (50)	22/31 (71)	8/11 (72.7)	7/8 (87.5)	6/11 (54.5)	1/1 (100)
Hyperoral behavior	18/27 (66.7)	4/8 (50)	3/4 (75)	9/9 (100)	2/6 (33.3)	18/31 (58.1)	6/11 (54.5)	5/8 (62.5)	7/11 (63.6)	0/1 (0)
Ritualistic behavior	25/27 (92.6)	8/8 (100)	4/4 (100)	9/9 (100)	4/6 (66.7)	24/31 (77.4)	6/11 (54.5)	7/8 (87.5)	10/11 (90.9)	1/1 (100)
Language features, N (%)										
Word-finding diff	21/27 (77.8)	8/8 (100)	3/4 (75)	6/9 (66.7)	4/6 (66.7)	25/31 (80.6)	10/11 (90.9)	4/8 (50)	10/11 (90.9)	1/1 (100)
Agrammatism	13/27 (48.1)	6/8 (75)	1/4 (25)	1/9 (11.1)	5/6 (83.3)	3/31 (9.7)	1/11 (9.1)	1/8 (12.5)	1/11 (9.1)	0/1 (0)
Imp gramm compr	12/27 (44.4)	6/8 (75)	1/4 (25)	0/9 (0)	5/6 (83.3)	8/31 (25.8)	4/11 (36.4)	2/8 (25)	2/11 (18.2)	0/1 (0)
Imp naming	21/27 (77.8)	7/8 (87.5)	1/4 (25)	8/9 (88.9)	5/6 (83.3)	21/31 (67.7)	7/11 (63.6)	4/8 (50)	9/11 (81.8)	1/1 (100)
Nonfluent speech	16/27 (59.3)	7/8 (87.5)	1/4 (25)	3/9 (33.3)	5/6 (83.3)	6/31 (19.4)	4/11 (36.4)	2/8 (25)	0/11 (0)	0/1 (0)
Imp repetition	14/27 (51.9)	6/8 (75)	0/4 (0)	3/9 (33.3)	5/6 (83.3)	6/31 (19.4)	4/11 (36.4)	1/8 (12.5)	1/11 (9.1)	0/1 (0)
Imp single-word compr	7/27 (25.9)	3/8 (37.5)	0/4 (0)	4/9 (44.4)	0/6 (0)	11/31 (35.5)	2/11 (18.2)	2/8 (25)	7/11 (63.6)	0/1 (0)
Other features, N (%)										
MND signs	1/27 (3.7)	1/8 (12.5)	0/4 (0)	0/9 (0)	0/6 (0)	3/31 (9.7)	1/11 (9.1)	2/8 (25)	0/11 (0)	0/1 (0)
Parkinsonism	23/27 (85.2)	6/8 (75)	3/4 (75)	8/9 (88.9)	6/6 (100)	15/31 (48.4)	8/11 (72.7)	3/8 (37.5)	3/11 (27.3)	1/1 (100)

CBD = corticobasal degeneration; compr = comprehension; diff = difficulties; FTLD-Tau = frontotemporal lobar degeneration with inclusions of the protein tau; FTLD-TDP = frontotemporal lobar degeneration with inclusions of the TDP-43 protein; imp = impaired; MAPT = FTLD-tau with *MAPT* gene mutation; PiD = Pick’s disease; MND = motor neuron disease; PSP = progressive supranuclear palsy; Type A/Type B/Type C/Type E = subtypes of FTLD-TDP

^a^Median and interquartile range are displayed for these variables

^b^Baseline features were defined as those occurring within the first three years from disease onset. 28 patients (FTLD-Tau = 12; FTLD-TDP = 16), with no visits available within the first three years, were excluded from baseline data

^c^Follow-up features were defined as those occurring after three years from disease onset. 31 patients (FTLD-Tau = 9; FTLD-TDP = 22), with no visits available after the first three years of disease, were excluded from follow-up data

To test the relationship between %AO measures of pathology burden and WM degeneration, adjacent luxol fast blue (LFB) stained sections from core regions were assessed for degeneration of WM by an experienced investigator (DJI) using a semi-quantitative scale based on the severity of disorganization and reduced density of white matter fibers in each slide (i.e. 0 = normal healthy WM; 1 = mild, 2 = moderate and 3 = severe WM degeneration; Supplementary Fig. 2) and compared to control tissue without neurodegeneration. Ratings were obtained blinded to neuropathological and clinical diagnosis and %AO data.

**Fig. 2:**
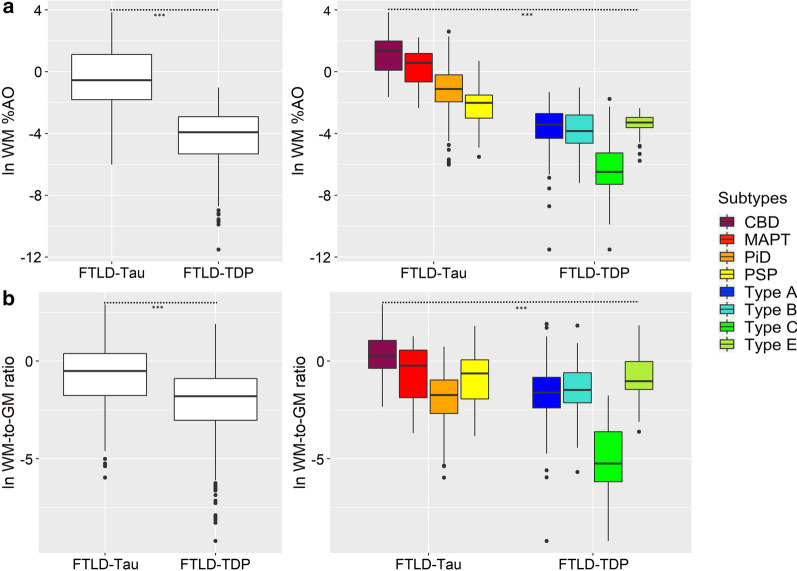
Comparisons of absolute and relative WM pathology burden between FTLD-Tau and FTLD-TDP groups and subtypes. Group differences between proteinopathies and their subtypes, across all regions examined, in **a** a digital measure of WM pathology burden (%AO, here with natural logarithmic transformation), and **b** a relative measure of WM pathology burden (WM-to-GM ratio, here with natural logarithmic transformation). Statistics were performed using a linear mixed-effects (LME) model to account for interdependency of multiple measurements from the same patient; all analyses found a significant effect of proteinopathy group or subtype on WM pathology (*p* < 0.001). Details of pairwise *post-hoc* comparisons between subtypes are shown in Supplementary Tables 4 and 6. Legend: %AO = percentage area occupied by pathology; CBD = corticobasal degeneration; FTLD-Tau = frontotemporal lobar degeneration with inclusions of the tau protein; FTLD-TDP = frontotemporal lobar degeneration with inclusions of the TDP-43 protein; MAPT = tau with *MAPT* gene mutation; PiD = Pick’s disease; PSP = progressive supranuclear palsy; Type A/Type B/Type C/Type E = subtypes of FTLD-TDP pathology; WM = white matter; WM-to-GM ratio = ratio of WM %AO to GM %AO

### Statistical analysis

Demographic and autopsy features were compared between proteinopathy groups (FTLD-Tau vs. FTLD-TDP) using independent samples t-tests for continuous variables. To compare continuous variables between multiple proteinopathy subtypes (FTLD-Tau: CBD, MAPT, PiD, PSP; FTLD-TDP: types A, B, C, E), we used analysis of variance (ANOVA), and when applicable, planned *post-hoc* pairwise t-tests with Bonferroni correction for multiple comparisons. Categorical variables (e.g. clinical features) were compared between proteinopathy groups and subtypes using Fisher’s Exact test. Quantitative pathology data (i.e. %AO measurements) were tested using parametric statistics after natural logarithmic (ln) transformation in order to meet the normality assumption of the parametric tests. Semi-quantitative pathology data in each region (i.e. LFB ordinal ratings) were compared between groups using non-parametric Mann–Whitney U analysis.

We used linear mixed-effects (LME) modeling with random intercepts for individual patients to account for interdependency of multiple measurements from the same patient and for missing data [34] for the following analyses: (1) comparing pathology measures between proteinopathies and their subtypes, (2) relating WM pathology burden (%AO) with WM degeneration scores (i.e. LFB ordinal ratings) across different regions, (3) comparing WM and GM pathology burden (%AO) between regions within FTLD-TDP and FTLD-Tau proteinopathies, their subtypes and clinicopathological groups. To compare WM pathology burden between distinct groups, we used a LME model with WM %AO as the dependent variable and proteinopathy group or subtype as a fixed-effect predictor of interest. We repeated this analysis using a relative measure of WM pathology burden, i.e. the ratio of WM %AO to GM %AO (WM-to-GM ratio), to account for potential morphological differences that could affect the comparison of absolute %AO between FTLD-Tau and FTLD-TDP pathologies. We also used a similar analysis to compare the severity of WM degeneration (i.e. LFB ordinal ratings) between FTLD-Tau and FTLD-TDP across different regions. To correlate WM pathology burden with WM degeneration on LFB staining, we used a LME model with WM %AO as dependent variable and LFB ordinal ratings as fixed-effect predictor of interest. To compare pathology burden between different regions, we used LME models with WM or GM %AO as dependent variable and region as fixed-effect predictor of interest. Based on least-square means resulting from these models, which are corrected for both random effects and covariates, we determined the regions of peak (i.e. greatest) pathology burden. The LME models included the following demographic/pathological variables as fixed-effect covariates to adjust for their potential influences on %AO measurements: brain region, brain hemisphere, proteinopathy group/subtype, disease duration and mutation status. For detailed information about each model, please see Supplementary Methods. To assess the effect of a multilevel categorical variable on the model, type III ANOVA with Satterthwaite approximation was employed. Planned *post-hoc* comparisons for significant LME outcomes were performed on LME-derived least-square means with Tukey correction for multiple comparisons. All analyses were two-sided with significance level set at 0.05, and were performed using R Statistical Software 4.0.0.

## Results

### Patients

Our cohort included 92 patients, 37/92 (40.2%) with FTLD-Tau (11 CBD, 5 MAPT, 12 PiD and 9 PSP) and 55/92 (59.8%) with FTLD-TDP (20 type A, 17 type B, 13 type C, 5 Type E). Of FTLD-Tau patients, 14/37 (37.8%) had PPA and 23/37 (62.2%) had bvFTD as primary clinical diagnosis. Of FTLD-TDP patients, 13/55 (23.6%) had PPA, whereas 42/55 (76.4%) had bvFTD. The relative frequency of clinical phenotypes was not different between FTLD-Tau and FTLD-TDP (p = 0.166), but differed between subtypes of each proteinopathy (p = 0.004) as shown in Table 1. PPA has relatively distinct clinical variants, which are associated with somewhat distinct regional patterns of brain atrophy [20]. Similar to known clinicopathological associations of these variants, in FTLD-Tau the majority had nonfluent variant PPA (naPPA, N = 10/14), while a minority had sematic variant PPA (svPPA, N = 1/14) or PPA with mixed features (mPPA, N = 3/14), while FTLD-TDP was mostly clinical svPPA (N = 8/13) and less commonly naPPA (N = 3/13) or mPPA (N = 2/13). Specific clinical features noted at baseline clinical visits (within 3 years from reported disease onset) and at longitudinal follow-up (> 3 years after disease onset) in each proteinopathy group and subtypes are reported in Table 2. FTLD-Tau and FTLD-TDP did not differ in demographic and autopsy-related features, yet proteinopathy subtypes showed differences in some of these features (Table 1). FTLD-Tau and FTLD-TDP differed in the frequency of genetic vs. sporadic cases (p < 0.001), as FTLD-Tau had fewer genetic cases (5/37, 13.5%) than FTLD-TDP (28/55, 50.9%). FTLD-TDP subtypes differed in the frequency of specific genetic mutations (p < 0.001) as shown in Table 1, reflecting known genetic associations with FTLD-TDP subtypes [37].

### Between-group comparisons of WM pathology burden

Qualitatively, FTLD-Tau WM pathology often consisted of moderate to severe amounts of diffuse tau-positive thread-like processes with dystrophic features along with frequent tau-positive coiled bodies in oligodendrocytes across subtypes as reported previously [12, 32]. In contrast, FTLD-TDP cases showed scant to moderate amounts of WM TDP-43 pathology across subtypes that were largely exclusive to oligodendrocytes, with variable axonal or other thread-like pathology in WM, consistent with previous reports [36, 53] (Fig. 1).

We used digital image analysis to measure the pathology burden of FTLD-Tau and FTLD-TDP in WM quantitatively, and we compared differences in WM pathology burden between proteinopathies and their subtypes (Fig. 2). When we directly compared FTLD-Tau and FTLD-TDP, we found greater absolute WM %AO in FTLD-Tau vs. FTLD-TDP (beta = 4.21, SE = 0.34, *p* < 0.001; Fig. 2a; Supplementary Table 3). This held true within each clinical phenotype of bvFTD (beta = 3.48, SE = 0.38, *p* < 0.001) and PPA (beta = 5.55, SE = 0.69, *p* < 0.001). When covarying for motor features (i.e. presence of parkinsonism and/or motor neuron disease signs at baseline or follow-up), there was no significant effect and the results were unchanged (data not shown). Additionally, WM pathology burden differed by proteinopathy subtype across FTLD-Tau and FTLD-TDP (F = 60.2, *df* = 7,83, *p* < 0.001). When we directly compared FTLD-Tau and FTLD-TDP subtypes using *post-hoc* pairwise comparisons, we found greater absolute %AO in WM as a unifying feature of FTLD-Tau subtypes compared to FTLD-TDP subtypes. CBD, MAPT, PSP and PiD all had greater WM %AO than FTLD-TDP type A, B and C (*p* ≤ 0.001; Fig. 2a; Supplementary Table 4). While CBD, MAPT and PiD had greater WM %AO than FTLD-TDP type E (*p* < 0.03), PSP did not (*p* > 0.05); this may in part be due to the small sample size in the type E subtype (N = 5). There was also some heterogeneity in WM pathology burden between distinct subtypes of the same proteinopathy. In FTLD-TDP, pairwise comparisons showed that FTLD-TDP type A, type B and type E had greater WM pathology burden than type C (*p* < 0.001). In FTLD-Tau, pairwise comparisons found that CBD had greater absolute WM pathology burden than PiD (*p* < 0.001) and PSP (*p* < 0.001). We also examined a relative measure of WM pathology, the WM-to-GM ratio, and similarly found greater relative WM pathology burden in FTLD-Tau vs. FTLD-TDP (beta = 2.09, SE = 0.33, *p* < 0.001; Fig. 2b; Supplementary Tables 5). The WM-to-GM ratio differed significantly between proteinopathy subtypes as well (F = 32.3, *df* = 7,84, *p* < 0.001), and showed similar trends to the absolute WM %AO comparisons (Fig. 2b; Supplementary Table 6), with the exception of PiD that had a lower relative WM burden, in part, due to more prominent GM pathology. While in FTLD-Tau 91/262 (35%) of the tissue sections had greater WM pathology burden than GM pathology burden (i.e. WM-to-GM ratio > 1); of these, 56/91 (62%) were tissue from CBD, 16/91 (18%) were from PSP, 14/91 (15%) were from MAPT and 5/91 (5%) were from PiD patients. In contrast, in FTLD-TDP a WM-to-GM ratio greater than 1 was only found in 21/363 (6%) of tissue samples, of whom 9 were from type A, 4 were from type B and 8 were from type E patients.

**Table 3:** LFB ratings of WM degeneration in core regions in FTLD-Tau vs. FTLD-TDP

	FTLD-Tau	FTLD-TDP	*P *value^#^
ACG	1 (1–2) N = 36	1 (0–2) N = 51	0.048
ANG	1 (0–1) N = 34	1 (0–1) N = 50	0.435
MFC	1 (1–3) N = 38	0 (0–2) N = 58	0.003
OFC	1 (0–3) N = 33	1 (0–2) N = 55	0.964
STG	1 (1–1) N = 34	1 (1–3) N = 48	0.282

Values depict median (interquartile range) of ordinal scores

ACG = anterior cingulate gyrus; ANG = angular gyrus; FTLD-Tau = frontotemporal lobar degeneration with inclusions of the tau protein; FTLD-TDP = frontotemporal lobar degeneration with inclusions of the TDP-43 protein; LFB = luxol fast blue; MFC = middle frontal cortex; OFC = orbitofrontal cortex; STG = superior temporal gyrus; WM = white matter

^#^LFB ordinal ratings were compared between pathology groups using Mann Whitney U analysis

Further, we were interested in testing whether the presence of a genetic mutation impacted WM pathology burden (Supplementary Table 7). We found that patients with a mutation across both proteinopathies had greater WM %AO than sporadic patients (beta = 1.02, SE = 0.34, p = 0.003) independent of proteinopathy subtype. In FTLD-TDP, WM pathology burden differed by specific genetic mutation group (F = 6.2, *df* = 3,49, *p* = 0.001). Greater WM %AO was found in *GRN* mutations compared to sporadic cases (*p* = 0.001) as well as in *C9orf72* cases compared to sporadic cases (*p* = 0.031). However, when covarying for proteinopathy subtype, this effect was less robust (F = 3.0, *df* = 3,48, *p* = 0.038), and the interaction between proteinopathy subtype and genetic mutation was not significant. In FTLD-Tau, genetic mutation did not have a significant effect on WM %AO (*p* > 0.05).

### WM degeneration in FTLD-Tau and FTLD-TDP

First, we compared the severity of a gold-standard measure of WM degeneration, i.e. ordinal ratings of myelin-stained LFB tissue between proteinopathy groups across regions (Supplementary Table 8). We found that there was greater overall severity of WM degeneration in FTLD-Tau compared to FTLD-TDP in the sampled core regions (beta = 0.36, SE = 0.18, *p* = 0.047). This effect was in part dependent on the brain region, which showed a significant interaction with pathology group when added to the model (F = 3.9, *df* = 4,341, *p* = 0.004). When we compared the severity of WM degeneration between FTLD-Tau and FTLD-TDP in different brain regions, we found that FTLD-Tau had greater WM degeneration scores in MFC (*p* = 0.003) and ACG (*p* = 0.048) than FTLD-TDP (Table 3), supporting our findings of more prominent WM degeneration in FTLD-Tau compared to FTLD-TDP.

**Fig. 3:**
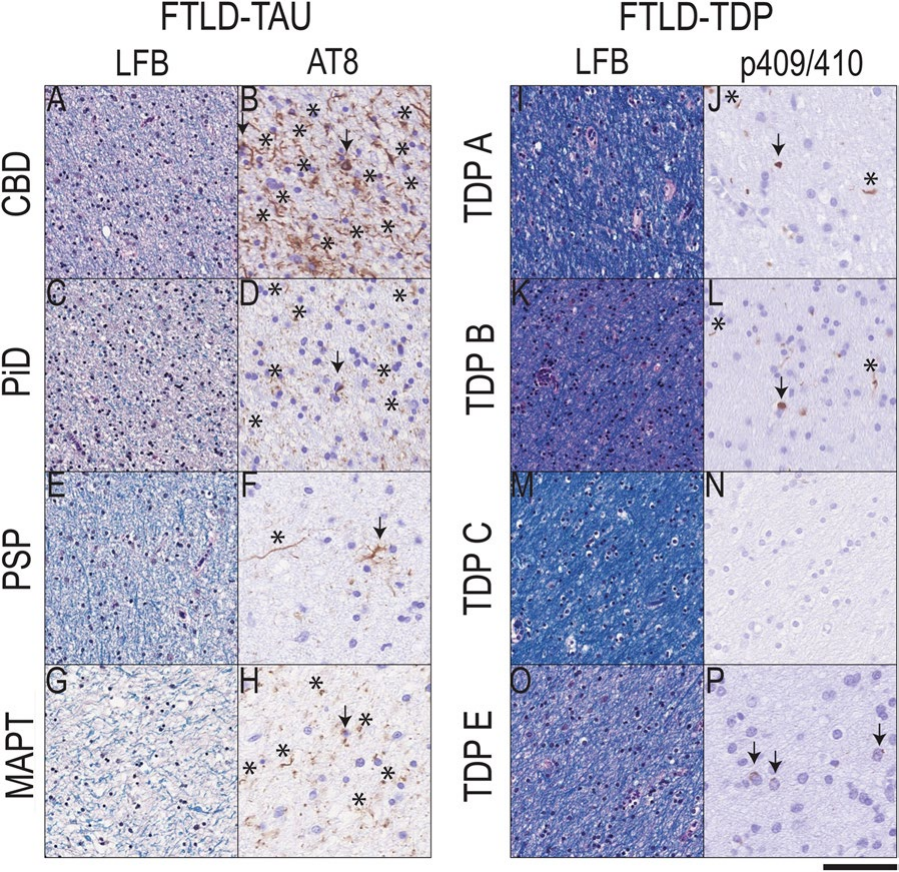
White matter degeneration and pathology burden in FTLD-Tau and FTLD-TDP. Photomicrographs depict representative images of group-level observations from myelin stain (LFB) and phosphorylated tau (AT8) in FTLD-Tau (A-H) or phosphorylated TDP-43 stained tissue in FTLD-TDP (I-P) from adjacent sections of middle frontal cortex white matter (WM). FTLD-Tau (CBD = A-B; PiD = C-D; PSP = E–F, MAPT = G-H) showed a greater frequency of tissue with moderate to severe WM degeneration with reduced LFB stain and disorganized fibers, which was accompanied by largely moderate to severe tau-positive axonal threads (asterisks) and glial tau inclusions (arrows); whereas FTLD-TDP (TDP subtype A = I-J; TDP subtype B = K-L, TDP subtype C = M–N, TDP subtype E = O-P) showed a greater frequency of normal to mildly degenerated WM on LFB stain with negligible or slightly reduced LFB stain and disorganization, and accompanied by relatively mild to moderate density of TDP-43 positive oligodendrocytes (arrows) and rare diffuse threads (asterisks). Scale bar = 50 µm. Legend: CBD = corticobasal degeneration; FTLD-Tau = frontotemporal lobar degeneration with inclusions of the tau protein; FTLD-TDP = frontotemporal lobar degeneration with inclusions of the TDP-43 protein; LFB = luxol fast blue; MAPT = tau with *MAPT* gene mutation; PiD = Pick’s disease; PSP = progressive supranuclear palsy; TDP A/TDP B/TDP C/TDP E = subtypes of FTLD-TDP pathology

Next, we tested the relationship between our digital measure of protein pathology burden and severity of WM degeneration in adjacent LFB-stained sections (Supplementary Table 9). Indeed, we found that our digital measure of pathology burden in WM reflected the severity of WM degeneration observed on LFB staining in both FTLD-Tau (beta = 0.32, SE = 0.10, *p* = 0.002) and FTLD-TDP (beta = 0.40, SE = 0.08, *p* < 0.001). In Fig. 3, we illustrate the correspondence between more frequent severe pathology burden and degeneration of WM fibers in middle frontal WM in FTLD-Tau compared to FTLD-TDP, also shown graphically as plots in Supplementary Fig. 3. Additionally, we found a positive significant association between LFB ordinal ratings and digitally measured GM %AO in FTLD-Tau (beta = 0.28, SE = 0.11, *p* = 0.013) and in FTLD-TDP (beta = 0.22, SE = 0.07, *p* = 0.002), although this was less strong than the relationship observed with WM %AO, suggesting that WM degeneration may in part be related to the severity of GM pathology.

### Regional distribution of WM and GM pathology burden in FTLD-Tau and FTLD-TDP and their subtypes

**Fig. 4:**
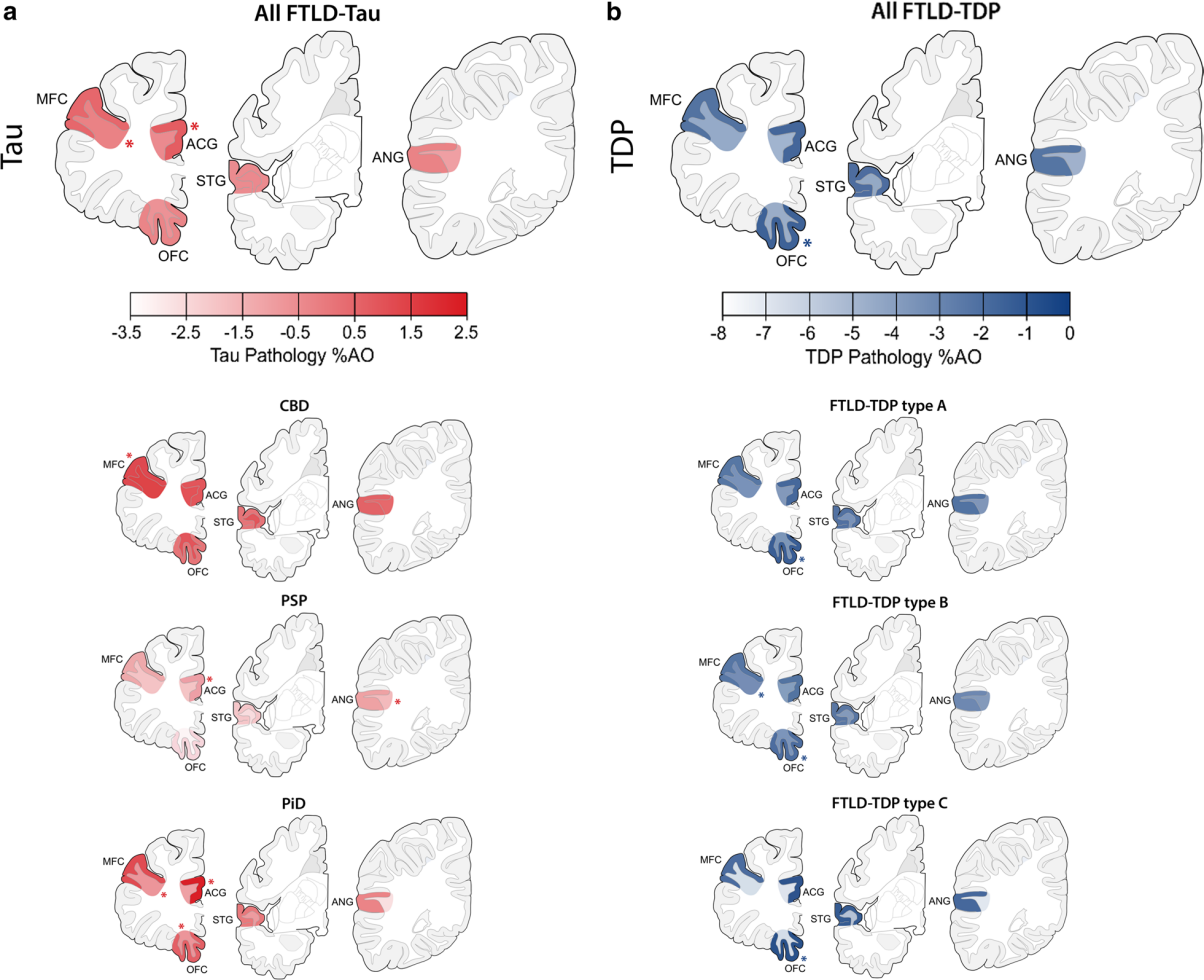
Regional distribution of WM and adjacent GM pathology burden in FTLD-Tau and FTLD-TDP. Plots portray the regional distribution of WM and GM pathology burden in FTLD-Tau (**a**) and FTLD-TDP (**b**) proteinopathies and their subtypes independent of clinical phenotype. The color scale represents least-square means of ln-transformed WM and GM %AO in each region derived from linear mixed-effects (LME) models adjusting for demographics. Asterisks denote areas of peak pathology burden. Legend: %AO = percentage area occupied by pathology; ACG = anterior cingulate gyrus; ANG = angular gyrus; CBD = corticobasal degeneration; FTLD-Tau = frontotemporal lobar degeneration with inclusions of the tau protein; FTLD-TDP = frontotemporal lobar degeneration with inclusions of the TDP-43 protein; GM = grey matter; MFC = middle frontal cortex; OFC = orbitofrontal cortex; PiD = Pick’s disease; PSP = progressive supranuclear palsy; STG = superior temporal gyrus; Type A/Type B/Type C = subtypes of FTLD-TDP pathology; WM = white matter

Finally, we investigated the regional pathology burden of tau and TDP-43 in WM as well as in adjacent GM, to assess whether FTLD-Tau and FTLD-TDP pathologies have distinctive patterns of pathology distribution independent of clinical phenotype (Fig. 4; Supplementary Tables 10 and 11).

In FTLD-Tau, pathology burden differed by region in WM (F = 3.4, *df* = 4,158, *p* = 0.010) as well as in GM (F = 7.0, *df* = 4,156, *p* < 0.001). In WM, the region of peak pathology burden was the MFC, whereas in GM the region of peak pathology burden was ACG (Fig. 4a). *Post-hoc* analysis in WM showed higher tau pathology burden in MFC that was greater than in ANG (*p* = 0.006). In GM, tau pathology burden was greater in ACG compared to ANG (*p* = 0.005), OFC (*p* = 0.002) and STG (p = 0.001), and also in MFC compared to STG (*p* = 0.020) and OFC (*p* < 0.05). In FTLD-TDP, pathology burden did not differ by region in WM (*p* = 0.297), but there were significant regional differences in GM (F = 8.2, *df* = 4,240, *p* < 0.001). The region of peak TDP-43 pathology burden in GM was the OFC (Fig. 4b). *Post-hoc* analysis in GM showed greater TDP-43 pathology burden in OFC compared to ANG (*p* < 0.001), MFC (*p* < 0.001) and STG (*p* = 0.017), as well as greater burden in ACG compared to ANG (*p* = 0.003) and MFC (*p* = 0.012).

All models in both FTLD-Tau and FTLD-TDP revealed a significant concurrent effect of pathology subtype on pathology burden in either GM or WM (*p* < 0.01). Therefore, we also examined the regional patterns of each pathology subtype and found largely similar patterns in FTLD-Tau subtypes (Fig. 4a; Supplementary Table 12) and FTLD-TDP subtypes (Fig. 4b; Supplementary Table 13) proteinopathies. In FTLD-Tau subtypes, regions of peak GM pathology burden were in the dorsolateral MFC (CBD) or the frontal paralimbic ACG (PSP, PiD); additionally, PSP had peak WM pathology burden in ANG, while PiD had peak WM pathology burden in OFC and MFC, which were both almost equally affected. In FTLD-TDP, peak GM pathology burden was found in the OFC consistently in subtypes A, B and C with minimal regional specificity of WM.

### Regional distribution of WM and GM pathology burden in clinico-pathological groups

**Fig. 5:**
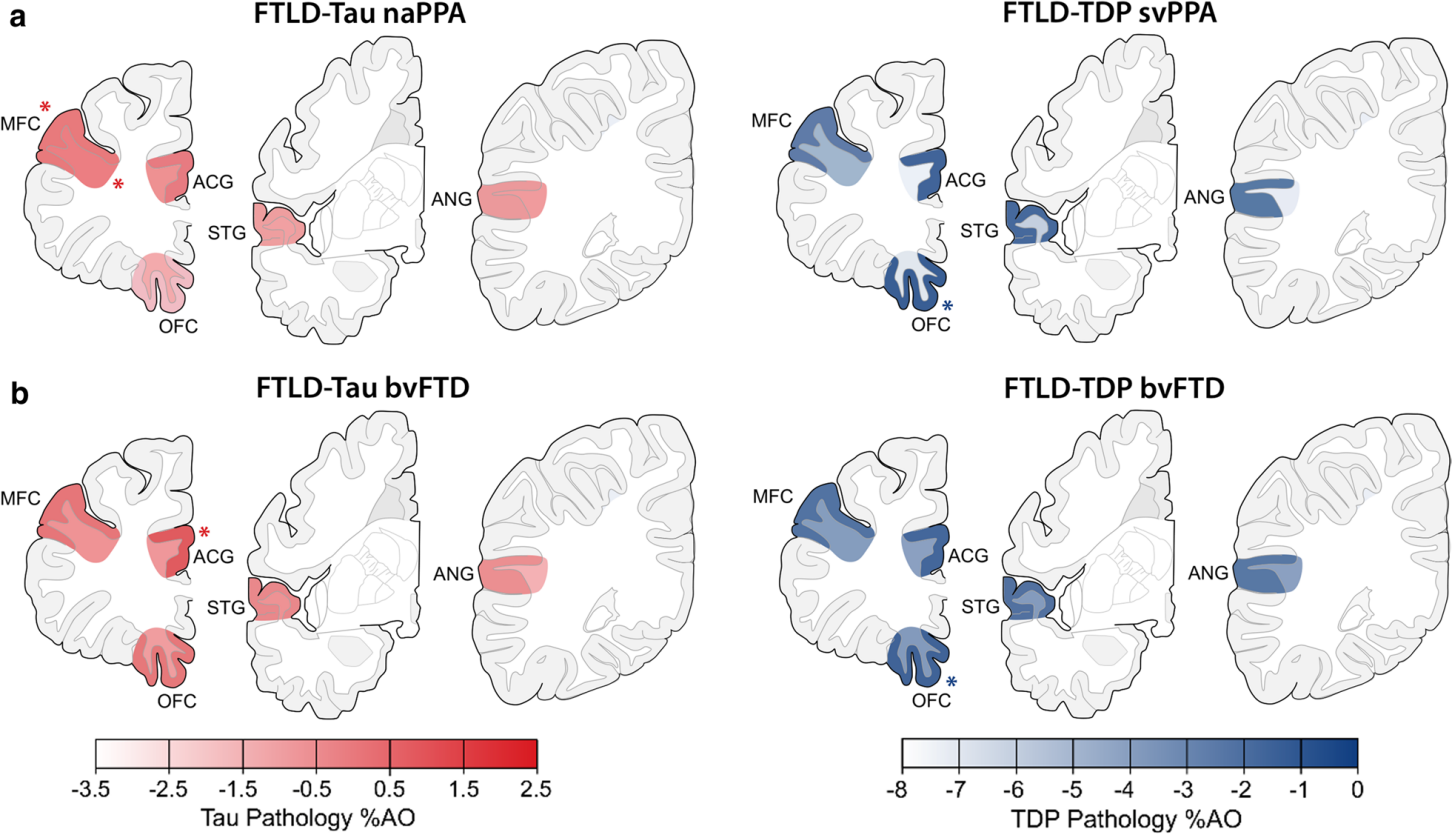
Regional distribution of WM and adjacent GM pathology burden in clinicopathological groups. Plots portray the regional distribution of WM and GM pathology burden in clinicopathological groups, i.e. **a** PPA with FTLD-Tau (nonfluent/agrammatic variant, naPPA) and PPA with FTLD-TDP (semantic variant, svPPA), and **b** bvFTD with FTLD-Tau and bvFTD with FTLD-TDP. The color scale represents least-square means of ln-transformed WM and GM %AO in each region derived from linear-mixed effects (LME) models adjusting for demographics. Asterisks denote areas of peak pathology burden. Legend: %AO = percentage area occupied by pathology; ACG = anterior cingulate gyrus; ANG = angular gyrus; bvFTD = behavioral variant of frontotemporal dementia; FTLD-Tau = frontotemporal lobar degeneration with inclusions of the tau protein; FTLD-TDP = frontotemporal lobar degeneration with inclusions of the TDP-43 protein; GM = grey matter; MFC = middle frontal cortex; naPPA = nonfluent/agrammatic variant of primary progressive aphasia; OFC = orbitofrontal cortex; STG = superior temporal gyrus; svPPA; semantic variant of primary progressive aphasia; WM = white matter

Finally, we investigated the regional distribution of absolute WM and GM pathology burden in FTLD-Tau and FTLD-TDP in each clinical phenotype to improve the understanding of clinical correlates of WM and GM pathology (Fig. 5; Supplementary Tables 14–15). We thus looked at naPPA with FTLD-Tau, svPPA with FTLD-TDP, bvFTD with FTLD-Tau and bvFTD with FTLD-TDP, based on well-established clinico-pathological associations with regional atrophy patterns [23, 48, 63].

In naPPA with FTLD-Tau, pathology burden differed by region in WM (F = 3.1, *df* = 4,40, *p* = 0.024), where the region of peak pathology burden was the MFC. Pathology burden also differed by region in GM (F = 8.0, *df* = 4,41, *p* < 0.001). The MFC was the region of peak pathology burden in GM as well (Fig. 5a). In contrast to PPA with FTLD-Tau, svPPA with FTLD-TDP did not show regional differences in pathology burden in WM (*p* = 0.267), but the pathology burden in GM differed by region (F = 9.6, *df* = 4,33, *p* < 0.001); the region of peak GM pathology burden was the OFC (Fig. 5a). Examination of the total PPA patients in each proteinopathy group, including those with mixed clinical features, found similar regional results (data not shown). Moreover, a sub-analysis in the left hemisphere of PPA patients showed similar regional findings (Supplementary Table 16). The anterior temporal lobe has been implicated in svPPA [45, 48], often associated with FTLD-TDP; however, our sample of svPPA with available tissue in this region (ventrolateral temporal cortex, i.e. VLT) was too small for analysis (N = 3).

In bvFTD with FTLD-Tau, unlike naPPA with FTLD-Tau, pathology burden did not differ by region in WM (*p* = 0.103), but it differed in GM (F = 3.3, *df* = 4,91, *p* = 0.015). The region of peak pathology burden in GM was the ACG (Fig. 5b). Likewise, in bvFTD with FTLD-TDP, pathology burden did not differ by region in WM (*p* = 0.631), but there were regional differences in GM (F = 5.3, *df* = 4,178, *p* < 0.001); as in svPPA with FTLD-TDP, the region of peak pathology burden in GM was the OFC (Fig. 5b). Further, we performed a sub-analysis in the subset of bvFTD patients with available data in extended regions (FTLD-Tau = 8, FTLD-TDP = 9) to look at pathology burden in the anterior insula (INS), a region implicated relatively early in bvFTD [62, 63] (Supplementary Table 17). In bvFTD with FTLD-Tau, INS had similar GM and adjacent WM pathology burden to ACG (*p* > 0.7), and had greater WM pathology burden than superior parietal lobe (SPL), a less affected region (*p* = 0.021). In bvFTD with FTLD-TDP, INS had similar GM and WM pathology burden to OFC (*p* > 0.9), and had greater burden than SPL in both GM (*p* = 0.026) and WM (*p* = 0.002).

## Discussion

In this large-scale digital histopathological comparative study of WM and adjacent GM pathology in a clinically well-defined FTLD autopsy cohort, we find that greater WM pathology burden and WM degeneration is a consistent neuropathological feature of tauopathies compared to TDP-43 proteinopathies (Figs. 1, 2; Table 3). We also find evidence of distinct patterns of regional pathology for both WM and GM in regional analyses of FTLD-Tau and FTLD-TDP (Fig. 4) and within each clinical bvFTD and PPA phenotype (Fig. 5). These findings suggest that there is a distinct cellular and regional signature of microscopic disease severity associated with each of these two discrete classes of FTLD proteinopathies that implicates both WM and GM, and that this appears to contribute to clinical phenotype. These rare comparative autopsy data have important implications for the understanding of clinicopathological mechanisms in FTD and for models of progressive neurodegeneration in the human brain that could inform *antemortem* diagnosis of underlying pathology through more detailed interrogation of both WM and GM in analyses of frontotemporal brain connectivity in vivo.

Our patient groups were representative of previous descriptive reports of FTD autopsy cohorts, with roughly 60% of the cohort pathologically diagnosed with FTLD-TDP and 40% with FTLD-Tau (Table 1; for comprehensive reviews please see [21, 26]). The two main proteinopathy groups had similar age of disease onset and disease duration. Clinical phenotypes had a similar frequency in FTLD-Tau and FTLD-TDP, and bvFTD was the most common clinical phenotype in both proteinopathy groups. Proteinopathy subtypes were somewhat more heterogeneous in regard to demographics and clinical presentation. Female sex was underrepresented in PSP, and patients with PSP were significantly older than other subtypes of FTLD-Tau and FTLD-TDP. Disease duration varied across subtypes; notably, PiD (mean ~ 11 years) and FTLD-TDP type C (mean ~ 9 years) had longer survival, while other tauopathies (mean ~ 6 years) and FTLD-TDP type B (mean ~ 6 years) and type E (mean ~ 2.5 years) had shorter disease duration. Our findings above largely align with previous literature [36, 65, 78], and suggest that specific forms of FTLD pathology may have somewhat distinct demographic and prognostic features. Thus, we used careful statistical modeling to account for demographic data, which could influence pathology measurements when comparing FTLD proteinopathies and their subtypes.

We focused on WM pathology since this has been understudied in FTLD. Similar to previous qualitative studies, we found prominent tau pathology in axonal threads, coiled-bodies within oligodendrocytes and astrocytic tau pathology, while FTLD-TDP pathology in WM was largely confined to oligodendrocytes [12, 32, 36, 53]. While these proteinopathies may have similar clinical presentations but different pathological substrates, the underlying patterns and severity of microscopic WM disease seldom have been compared directly. Here, using validated digital histopathological methods, we found greater severity of WM pathology in FTLD-Tau subtypes compared to FTLD-TDP subtypes (Fig. 1). Thus, we found consistent evidence for greater severity of WM pathology as a unifying feature of tauopathies. There was some heterogeneity within proteinopathy groups that reflects previous qualitative studies, with greatest WM tau pathology burden in CBD compared to other tauopathies [32], and particularly minimal WM TDP-43 pathology burden in FTLD-TDP type C that was lower than the other TDP subtypes [36, 53], including type E [35]. In FTLD-TDP, patients with *GRN* and *C9orf72* mutations had greater WM pathology burden than sporadic FTLD-TDP, although these contrasts were less robust after co-varying for pathological subtype. Indeed, FTLD-TDP type A has been associated with greater WM pathology in oligodendrocytes and axonal threads compared to other subtypes [36], and FTLD-TDP with *GRN* mutations (most commonly associated with FTLD-TDP type A) has been described to have significant WM TDP-43 pathology [28]. But even in these subsets of FTLD-TDP, the severity of WM pathology was less than that found in FTLD-Tau. Our finding of greater WM pathology in FTLD-Tau compared to FTLD-TDP remained robust when looking at a ratio of WM to adjacent GM pathology burden to account for the relative abundance of pathology in WM (Fig. 2b).

Indeed, we found that a sizeable proportion of FTLD-Tau tissue (35%) had greater %AO in WM than in adjacent GM, whereas this was extremely rare in FTLD-TDP tissue (6%). These data suggest that even with advanced disease severity, TDP-43 pathology is relatively confined to the GM, while FTLD-Tau has additional WM pathology that may develop independently and even exceed the severity of GM pathology. Concordantly, *postmortem* observations in ALS with relatively focal TDP-43 pathology find WM TDP-43 pathology limited to oligodendrocytes in close proximity to degenerating axons from motor nuclei [5, 7], while others find no evidence of deep corticospinal tract TDP-43 pathology in oligodendrocytes, suggesting that WM pathology may contribute minimally to disease severity in ALS [14, 31].

While it is difficult to make inferences about the spread of pathology from cross-sectional autopsy data, animal and cell model data of cell-to-cell transmission provide important insights into the interpretation of our findings. Injections of brain lysates from human brains with tauopathies result in the propagation of distinct morphologies of neuronal and glial tau pathology in both transgenic [3, 8] and wild-type mice [51] with strain-like properties. Moreover, oligodendrocytes alone can propagate tau pathology both in cell and animal models [50], suggesting that the high severity of tau pathology in WM observed in our study may contribute in part to the cortical spread of pathology. There is comparatively limited model system data for TDP-43 propagation, but recent studies suggest that TDP-43 pathogenic species can also be transmitted between cells [15, 55]. Moreover, lysates from human brains with FTLD-TDP induce TDP-43 pathology in transgenic animals, but TDP-43 pathology in oligodendrocytes is relatively mild and occurs only at a later stage [57]. One possible interpretation of our data of relatively low WM pathology in FTLD-TDP is that TDP-43 pathological aggregation in WM oligodendrocytes may occur together with axons from degenerating neurons as a relatively late phenomenon. This hypothesis may also be supported by the lack of ubiquitin-reactivity in TDP-43 pathology in oligodendrocytes, which is a feature seen in more mature TDP-43 neuronal inclusions [53].

Examination of regional patterns of pathology revealed a divergent anatomic distribution of both WM and adjacent GM pathology in FTLD-Tau compared to FTLD-TDP. In FTLD-Tau, we observed the most prominent WM pathologic burden in the paralimbic mediofrontal and dorsolateral frontal regions, and GM %AO was greatest in dorsolateral frontal cortex (Figs. 4, 5). While this pattern appeared largely consistent across subtypes of FTLD-Tau, we did find that PSP has increased WM pathology burden in the parietal lobe. Detailed reports on the regional distribution of PSP tauopathy have largely focused on GM, but they highlight relative greater tau burden in frontoparietal regions compared to temporal neocortex [33, 75]. In vivo imaging of WM suggests changes in the superior longitudinal fasciculus and other fronto-parietal tracts in PSP [68, 72] and CBD [13]. Thus, our *postmortem* findings here may reflect tau involvement in these long-range WM tracts. PiD, the 3R-predominant tauopathy, had a slightly different tau distribution, which was most prominent in the GM of medial paralimbic ACG and WM adjacent to the orbitofrontal and middle frontal cortices. Indeed, rare pre-symptomatic autopsies [46, 66, 70] and analysis of mature tau conformations in PiD suggest a potential paralimbic origin of pathology [24], including the medial temporal lobe, anterior insula and anterior cingulate gyrus, which may reflect the patterns of tau pathology observed here. Moreover, PiD patients with overall mild disease have tau pathology in the ventral and dorsolateral frontal regions [24, 66], which may suggest spread of disease from paralimbic to adjacent frontal and temporal areas. Indeed, in vivo imaging finds prominent degeneration of the frontal cortex and frontal WM association tracts in autopsy-confirmed PiD [24]. Thus, while we did observe some regional heterogeneity between 3 and 4R tauopathies, WM in the medial and dorsolateral frontal regions and their associated GM regions appear preferentially diseased.

These findings of WM regional heterogeneity in FTLD-Tau stand in contrast to our observations of a relatively homogeneous distribution of WM pathology in FTLD-TDP. Thus, in FTLD-TDP, there was no apparent differential regional distribution of WM pathology. This finding must be interpreted cautiously due to the relatively limited sampling, however this may not fully explain our null result because we observed heterogeneity in the anatomic distribution of GM pathology in these same set of regions in FTLD-TDP. The OFC is an area that we previously found to have prominent TDP-43 GM pathology even in very mild disease samples [6], suggesting that it may be, along with other frontoinsular regions [49], an early locus of TDP-43 pathology in FTD. Additional work is needed to determine the basis for the relatively homogenous WM findings in FTLD-TDP.

The precise relationship between pathological protein deposition and WM degeneration in the human brain is currently unclear. Thus, we included blinded ordinal ratings of WM degeneration using adjacent tissue stained with LFB to support our digital analyses of pathology burden. We found group-level differences between FTLD-Tau and FTLD-TDP, as well as a correlation of WM degeneration with WM pathology burden for both tau and TDP-43. We also observed some heterogeneity in the regional distribution of WM degeneration, with the most prominent WM degeneration in dorsolateral and paralimbic frontal regions in FTLD-Tau, greater than in FTLD-TDP (Table 3). The regional heterogeneity in WM degeneration shown with LFB corresponds to the regional heterogeneity for pathologic burden seen in FTLD-Tau. While gold-standard ordinal ratings of WM degeneration are less granular than digital metrics, they constitute a reliable and validated reference method for the assessment of WM degeneration, while high-throughput digital methods for a large-scale assessment currently lack validation. A limitation of LFB is that is does not fully differentiate between WM degeneration due to intrinsic WM pathology and WM degeneration due to axonal loss from degenerating neurons in the GM. When we looked at the correlation of LFB ordinal ratings with WM and GM %AO, we found a significant correlation in both analyses that was stronger in WM than in GM. Based on this, we cannot exclude a contribution of axonal loss to the severity of WM degeneration. Future work should examine the independent contributions of axonal loss and myelin integrity in more detail to better characterize WM degeneration in FTLD. Nonetheless, our observations of protein pathology burden in WM and ordinal ratings of resultant WM degeneration both suggest differential involvement of WM tracts between clinically similar FTLD proteinopathies, with greater severity of WM pathology in FTLD-Tau compared to FTLD-TDP. Together with regional heterogeneity in the anatomic distribution of GM pathology in FTLD-Tau, the heterogeneous patterns of WM disease may influence the clinical consequences of these proteinopathies in the degradation of large-scale neurocognitive networks.

Indeed, we observed that the different neuroanatomic distributions of WM disease interacted with the clinical phenotypes most often associated with FTLD-spectrum pathology. We observed relatively severe WM pathology in the dorsolateral frontal region of FTLD-Tau with naPPA (Fig. 5a), as well as peak GM pathology burden in MFC of FTLD-Tau with naPPA. The dorsolateral frontal region has been implicated in naPPA together with inferior frontal regions [23]; the prominent severity of pathology in MFC WM as well as GM may contribute to this relatively distinct clinical variant of naPPA associated with FTLD-Tau. A recent study of FTLD-Tau examining deep WM tracts found subtle differences in tau pathology between subcortical WM tracts that were associated with behavioral or motor clinical phenotypes during life [30], further suggesting that WM disease may impact the clinical presentation of tauopathies. While we did not observe an influence of motor features on our pathology data in this study focused on dementia presentations of FTLD, future work should contrast bvFTD and PPA with motor phenotypes of ALS, PSP and CBS. In bvFTD associated with FTLD-Tau pathology, we observed greater GM pathology burden in ACG. ACG is a limbic region within the paralimbic salience network associated with bvFTD [63, 64] and it appears to be associated with apathy [40] and limited self-appraisal [22, 39]. Thus, both WM heterogeneity and GM heterogeneity appeared to contribute to clinical phenotype in FTLD-Tau.

In contrast, we did not observe specific associations of clinical phenotype with regional WM pathology burden in FTLD-TDP, with relatively homogeneous regional distribution of WM TDP-43 pathology. While we found some heterogeneity in the anatomic distribution of GM pathologic burden in FTLD-TDP, with peak GM pathology in OFC, this was regardless of clinical phenotype. OFC has been implicated in behavioral features in bvFTD [27, 71] and has been linked to semantic language deficits in these patients [10, 11]. Prominent atrophy in anterior temporal cortex has been associated with semantic impairments [45, 48] found in svPPA, often associated with FTLD-TDP proteinopathy, as well as PiD tauopathy. Since our core pathology sampling did not include the anterior temporal cortex, we had insufficient data to analyze this region and we thus cannot rule out the possibility of regional differences between clinical phenotypes or proteinopathy subtypes in this anterior temporal region. Yet, we have shown previously that pathologic severity in the anterior temporal lobe and in the orbitofrontal cortex in svPPA may be tightly related to each other, due to regional proximity, involvement in the same language network as well as functional connections via the uncinate fasciculus [18].

Thus, we find distinctive regional patterns of WM and GM pathology between proteinopathies but also within proteinopathies in association with clinical phenotype. In vivo imaging studies find patterns of cortical atrophy corresponding to functional connectivity patterns that define the salience network in bvFTD [63, 64] and the left-hemispheric language network in PPA [9, 18]. However, most studies lack regional autopsy findings, so it is currently unclear how distinct proteinopathies disrupt these cognitive networks and result in the clinical presentations of bvFTD or PPA. Consistent with our histopathology data, rare autopsy-confirmed imaging studies find subtle variations in atrophy patterns within and between brain hemispheres when comparing FTLD proteinopathies in each clinical phenotype [18, 27, 56, 60, 67, 73, 74], but this work offers limited consideration of WM disease. We add to this literature by noting the specific involvement of dorsolateral frontal WM pathology in FTLD-Tau with PPA, and the distinct microscopic neuroanatomic patterns of GM pathology in bvFTD and PPA depending on the underlying proteinopathy. Moreover, our findings based on fine-grained digital measurements also substantiate the hypothesis that distinct proteinopathies may perturb divergent cellular and regional “nodes” in the same salience or language network to ultimately cause somewhat similar clinical phenotypes [42, 61]. Clarifying these important issues will require future work in larger, comparative datasets with more high-density cortical and subcortical sampling across hemispheres integrated with in vivo imaging and informed by animal or cellular model data, in order to fully understand the pathogenesis and progression of disease from cellular to macroscopic regional levels.

While the rigorously validated digital histopathological approach and the use of a large well-annotated cohort were the main strengths of this study, some limitations should be considered. We studied relatively rare pathologies in patients with well-characterized but relatively rare *antemortem* clinical syndromes, and accounted for our relatively small samples by performing sub-analyses within pathology subtypes or statistically accounting for pathological subtype in our analyses. Nevertheless, additional work is needed with larger samples. Our sampling extends beyond traditional neuropathological sampling optimized for the diagnosis of AD [47], but we have relatively limited availability of regional sampling compared to the entire set of regions that comprise whole-brain in vivo imaging approaches. Our 6 µm sections provide limited depth of view of anatomical structures, such as scant TDP-43 positive axonal threads that are more readily observable in thick-section preparations [6, 7], but they do allow for large-scale quantitative measurements collected for this study (> 600 slides) that are prohibitive using a traditional stereological approach. While our measurements of WM pathology burden adjacent to cortical GM provide novel evidence for WM involvement in FTLD, they only approximate deep WM tracts that require visualization through dedicated sampling. We focused our regional analyses on the most typical clinical PPA variants in each proteinopathy (i.e. naPPA in FTLD-Tau and svPPA in FTLD-TDP) [67] since the less commonly associated clinical forms of PPA were limited in each proteinopathy group (Table 1). Proteinopathy subtypes may have different morphological features and anatomical distribution that may influence our comparisons of FTLD-Tau vs. FTLD-TDP; however, we rigorously accounted for subgroup differences in our models, and we validated our findings with additional (relative) measures of pathology, such as the WM-to-GM ratio and LFB ordinal ratings. While we accounted for genetic status statistically in our main analyses, we could not account for specific point mutations due to the limited sample size. Future work in larger cohorts and with specific stains (e.g. axonal stains) will facilitate direct comparisons of pathology within more fine-grained clinico-pathological groups and shed light on additional aspects of WM degeneration in FTLD. Finally, we studied an autopsy cohort of a relatively rare disorder from a tertiary referral center, thus there may be inherent referral biases that limit the interpretation of the relative frequencies of each proteinopathy and clinical phenotype presented in Table 1.

In summary, we find that a high level of WM pathology is a unifying feature of tauopathies, and that the heterogeneous anatomic distribution of WM pathologic burden may influence the clinical presentation of tauopathies, with prominent dorsolateral frontal WM tau as a distinguishing feature of the clinical syndrome of naPPA. This is in addition to the relatively heterogeneous anatomic distribution of GM pathology associated with naPPA compared to bvFTD in FTLD-Tau. This pathologic profile is distinct from the pattern of pathology observed in TDP-43 proteinopathies, where we find overall limited WM pathology, which is distributed in a relatively homogeneous manner across regions, but elevated GM pathology in ventromedial frontal regions that is apparent regardless of the clinical syndrome.

## Supplementary Information

**Additional file 1.**

## Data Availability

Data will be made available by the corresponding author upon reasonable request.
