# Supplementary material for: Frontotemporal lobar degeneration proteinopathies have disparate microscopic patterns of white and grey matter pathology
Source: Acta Neuropathol Commun. 2021 Feb 23;9:30. doi: 10.1186/s40478-021-01129-2 (PMC7901087; doi:10.1186/s40478-021-01129-2)
Supplement: Supplementary file 1 — Additional file 1. [file 40478_2021_1129_MOESM1_ESM.pdf]

Acta Neuropathologica Communications – **Online supplement for:**

Frontotemporal lobar degeneration proteinopathies have disparate microscopic patterns of white and grey matter pathology

Lucia A.A. Giannini[1,2,3], Claire Peterson[1,2], Daniel Ohm[1,2], Sharon X. Xie[4], Corey T. McMillan[2], Katya Raskovsky[2], Lauren Massimo[2], EunRah Suh[5], Vivianna M. Van Deerlin[5], David A. Wolk[6,7], John Q. Trojanowski[5,6], Edward B. Lee[6,8], Murray Grossman[2] and David J. Irwin[1,2]

[1]Digital Neuropathology Laboratory, Department of Neurology, Perelman School of Medicine, University of Pennsylvania, PA 19104, USA
[2]Penn Frontotemporal Degeneration Center, Department of Neurology, Perelman School of Medicine, University of Pennsylvania, PA 19104, USA
[3]Alzheimer Center, Department of Neurology, Erasmus University Medical Center, Rotterdam, The Netherlands
[4]Department of Biostatistics, Epidemiology and Informatics, Perelman School of Medicine, University of Pennsylvania, PA 19104, USA
[5]Center for Neurodegenerative Disease Research, Department of Pathology and Laboratory Medicine, Perelman School of Medicine, University of Pennsylvania, PA 19104, USA
[6]Alzheimer's Disease Center, Department of Pathology and Laboratory Medicine, Perelman School of Medicine, University of Pennsylvania, PA 19104, USA
[7]Department of Neurology, Perelman School of Medicine, University of Pennsylvania, PA 19104, USA
[8]Translational Neuropathology Research Laboratory, Department of Pathology and Laboratory Medicine, Perelman School of Medicine, University of Pennsylvania, PA 19104, USA

Running title: Regional microscopic pathology in FTLD

Please send correspondence to:
David J. Irwin
Frontotemporal Degeneration Center (FTDC)
University of Pennsylvania Perelman School of Medicine
Hospital of the University of Pennsylvania
3600 Spruce Street, Philadelphia, PA 19104
(215)-662-7682
dirwin@pennmedicine.upenn.edu

Supplementary materials: 1 Supplementary Methods, 3 Figures, 17 Tables

Supplementary Methods

Clinical chart review

Clinical features were extracted from patients' medical charts in a standardized manner by a clinician and/or researcher experienced in cognitive neurology (DJI, LAAG). Clinical notes from neurological visits were available in 89/92 (96.7%) patients of the total cohort (FTLD-Tau = 36, FTLD-TDP = 53). Three patients had only limited clinical information available, obtained from nursing homes, and were thus excluded from clinical analyses. The median number of available visits per patient was 3 (interquartile range [IQR] 2-8), with a slight difference between FTLD-Tau (median 5, IQR 2-10) and FTLD-TDP (median 3, IQR 2-5, p = 0.027). We collected features of behavior and language, characteristic of bvFTD and PPA respectively, and motor features. Behavioral features, recorded based on the clinical history or on direct observations of the clinician, were the following: apathy/inertia (i.e. loss of interest, drive or motivation), social disinhibition (inappropriate or impulsive behavior, or loss of social manners / decorum), loss of empathy (i.e. diminished understanding for other people's feelings or loss of personal warmth), hyperoral behavior (i.e. altered eating preferences or binge eating), ritualistic behavior (i.e. including complex and simple rituals or stereotypic speech). Language features, recorded based on observations of the clinician, were the following: word finding difficulties (i.e. word-finding pauses and/or circomlocutions in spontaneous speech), agrammatism (i.e. reduced grammatical structure of speech and/or grammatical errors in spontaneous speech), impaired grammatical comprehension (i.e. impaired understanding of a grammatically complex sentence), impaired naming (i.e. difficulty on confrontation naming tasks), nonfluent speech (i.e. slow and/or effortful speech), impaired repetition (i.e. difficulty repeating a sentence), impaired single-word comprehension (i.e. inability to comprehend words or objects). Motor features, recorded based on the observations of the clinician, were: motor neuron disease signs (i.e. upper motor neuron signs, i.e. spasticity and hyperreflexia, and/or lower motor neuron signs, i.e. fasciculations and muscle atrophy), parkinsonism (i.e. bradykinesia and/or rigidity; other parkinsonian features were considered supportive evidence and almost always occurred in conjunction with bradykinesia and/or rigidity; postural instability or impaired ocular motility alone were not considered sufficient evidence for parkinsonism). Features were categorized as either impaired / present or spared / absent, and the year of appearance of a clinical feature was recorded relative to the time of disease onset. Next, the frequency of clinical features was estimated at baseline (i.e. 0-3 years from disease onset), and at follow-up (i.e. >3 years from disease onset). 28 patients (FTLD-Tau = 12; FTLD-TDP = 16) had no visits available within the first three years, and were thus excluded from baseline data; 31 patients (FTLD-Tau = 9; FTLD-TDP = 22) had no visits available after the first three years, and were thus excluded from follow-up data.

Statistical analysis: between-group comparisons of WM pathology burden

We compared the absolute severity of WM pathology burden (WM %AO) between FTLD-Tau and FTLD-TDP using a LME model with WM %AO as dependent variable and proteinopathy group (FTLD-Tau vs. FTLD-TDP) as fixed-effect predictor of interest. Fixed-effect covariates were brain region, brain hemisphere, disease duration and mutation status. We also calculated a ratio of WM %AO to GM %AO (i.e. WM-to-GM ratio) to obtain a relative measure of WM pathology burden. We compared the relative severity of WM pathology between FTLD-Tau and FTLD-TDP using a similar LME model, with WM-to-GM ratio as dependent variable, proteinopathy group as predictor of interest and the same covariates. For these and the following analyses, we applied a natural logarithmic transformation to digital pathology measures (%AO, WM-to-GM ratio) in order to meet the assumptions of LME modeling (i.e. normality of residuals, homogeneity of variance).

Further, we were interested in comparing absolute WM pathology between distinct proteinopathy subtypes. For this purpose, we used a LME model with WM %AO as dependent variable and proteinopathy subtype as fixed-effect predictor of interest (FTLD-Tau: CBD, MAPT, PiD, PSP; FTLD-TDP: type A, B, C, E). Fixed-effect covariates were brain region, brain hemisphere, disease duration and mutation status.

We were also interested in looking at the effect of genetic mutations on the severity of WM pathology. We first tested the effect of genetic mutations across both proteinopathy groups using the dichotomous variable of mutation status (genetic vs. sporadic), and then within each proteinopathy group comparing specific mutations (*C9orf72* vs. *GRN* vs. *TBK1* vs. sporadic in FTLD-TDP; *MAPT* vs. sporadic in FTLD-Tau). Fixed-effect covariates were brain region, brain hemisphere, proteinopathy subtype and disease duration.

Statistical analysis: LFB ordinal ratings of WM degeneration and their relation to WM pathology Burden

First, we compared the severity of WM degeneration between FTLD-Tau and FTLD-TDP across all regions using a LME model with LFB ordinal ratings as dependent variable, proteinopathy group as fixed-effect predictor of interest, covarying for brain region, brain hemisphere, mutation status and disease duration. As the effect of pathology seemed to vary between different brain regions, we added an interaction term (proteinopathy group * brain region) to the model, and we assessed the overall effect of the interaction with a LME-based ANOVA analysis. Subsequently, we looked at comparisons of LFB ordinal ratings in each region using non-parametric Mann Whitney U analysis.

Next, we tested the relation between WM pathology burden (WM %AO) and LFB ordinal ratings of WM degeneration across multiple regions in FTLD-Tau and FTLD-TDP. We used a LME model with WM %AO as dependent variable and LFB ordinal ratings as fixed-effect predictor of interest. Fixed-effect covariates were brain region, brain hemisphere, proteinopathy subtype, disease duration and mutation status

(in FTLD-TDP only). In FTLD-Tau, since *MAPT*-mutated cases were grouped as a distinct proteinopathy subtype, we could not separately covary for mutation status due to collinearity.

Statistical analysis: regional distribution of WM and GM pathology burden in FTLD-Tau and FTLD-TDP and clinicopathological groups

We used a LME model to test whether WM and adjacent GM pathology burden differed in regional distribution in both proteinopathies and clinicopathological groups. We first looked separately at both WM and GM %AO within FTLD-Tau and FTLD-TDP proteinopathy groups. The models included either WM or GM %AO as dependent variable and brain region as fixed-effect predictor of interest. Fixed-effect covariates were brain hemisphere, proteinopathy subtype, disease duration and mutation status (in FTLD-TDP only). In case of a significant main effect of region using type III ANOVA, we performed pairwise comparisons of WM or GM %AO between regions using a planned *post-hoc* analysis with Tukey correction for multiple comparisons. All regional analyses were performed using data from five core regions (i.e. ACG, ANG, MFC, OFC, STG), which were sampled in the entire cohort.

We performed a sub-analysis within proteinopathy subtypes (FTLD-Tau: CBD, MAPT, PiD, PSP; FTLD-TDP: Type A, Type B, Type C, Type E). For this, we used a model with WM or GM %AO as dependent variable and brain region as fixed-effect predictor of interest within each subtype. Fixed-effect covariates were brain hemisphere and disease duration.

To examine the relation between pathology distribution and clinical phenotypes, we performed a subanalysis to assess the regional distribution of WM and GM pathology burden in nonfluent/agrammatic PPA (naPPA) with FTLD-Tau, in semantic variant PPA (svPPA) with FTLD-TDP, and in bvFTD with either FTLD-Tau or FTLD-TDP. Similar to the prior analysis, these analyses used separate models for WM and GM %AO as dependent variables and brain region as fixed-effect predictor of interest. Fixed-effect covariates were brain hemisphere and disease duration.

Supplementary Figure 1. Validation of GM and WM %AO measurements in FTLD-Tau and FTLD-TDP based on gold-standard ordinal ratings of Tau/TDP-43 pathology

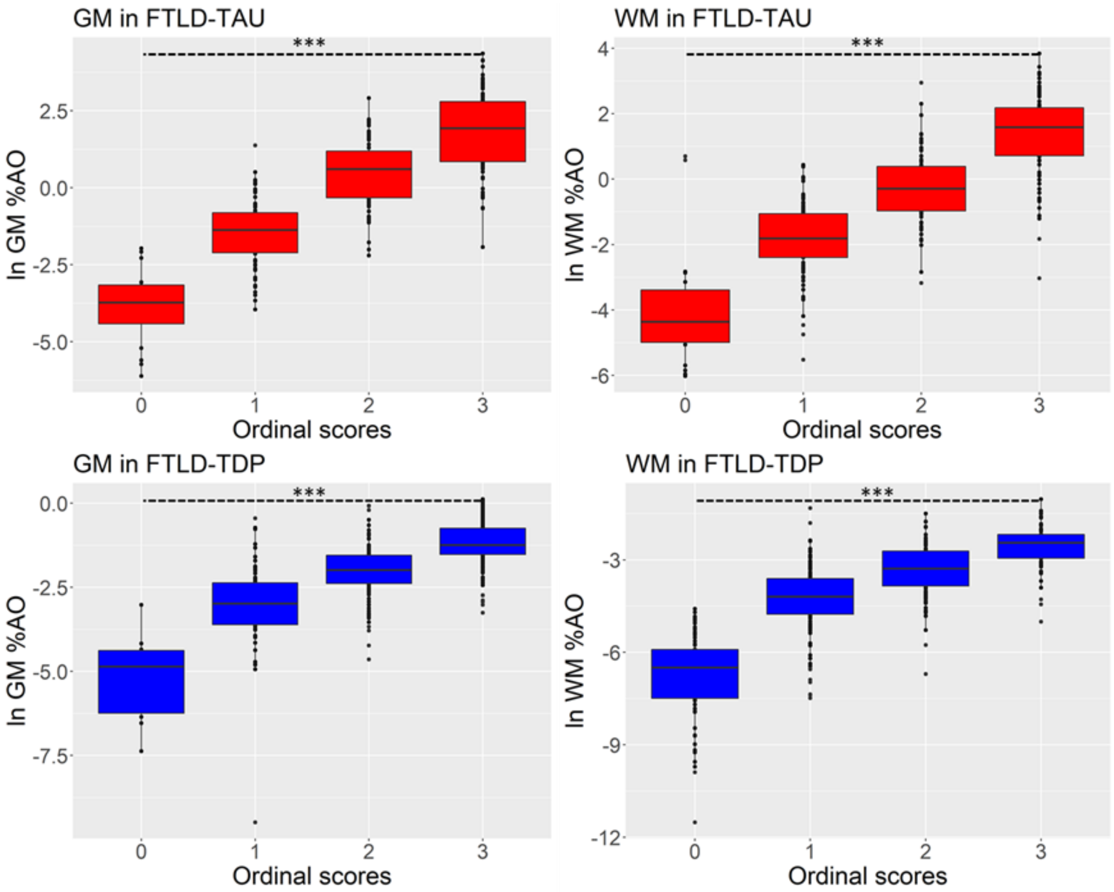

Plots portray the relationship between conventional ordinal ratings of pathology (i.e. 0-3 scores for each proteinopathy) and digitally acquired %AO scores after natural logarithmic transformation in FTLD-Tau (above) and FTLD-TDP (below). ANOVA analysis finds that ordinal scores are significantly associated with digital ln %AO scores in FTLD-Tau GM ($F = 495$, df = 1,266, $p < 0.001$) and WM ($F = 448$, df = 1,267, $p < 0.001$), and in FTLD-TDP GM ($F = 308$, df = 1,340, $p < 0.001$) and WM ($F = 468$, df = 1,346, $p < 0.001$). In *post-hoc* pairwise comparisons, we found a significant difference in ln %AO scores between each ordinal-score group in both FTLD-Tau and FTLD-TDP GM/WM ($p < 0.001$, Bonferroni-corrected), suggesting that digital AO% measurements accurately reflected the severity of pathology consistently with gold-standard ordinal ratings.

Legend: %AO = percentage area occupied by pathology; FTLD-Tau = frontotemporal lobar degeneration with inclusions of the tau protein; FTLD-TDP = frontotemporal lobar degeneration with inclusions of the TDP-43 protein; GM = grey matter; WM = white matter.

Supplementary Figure 2. Exemplary images of LFB-based ordinal ratings of white matter degeneration

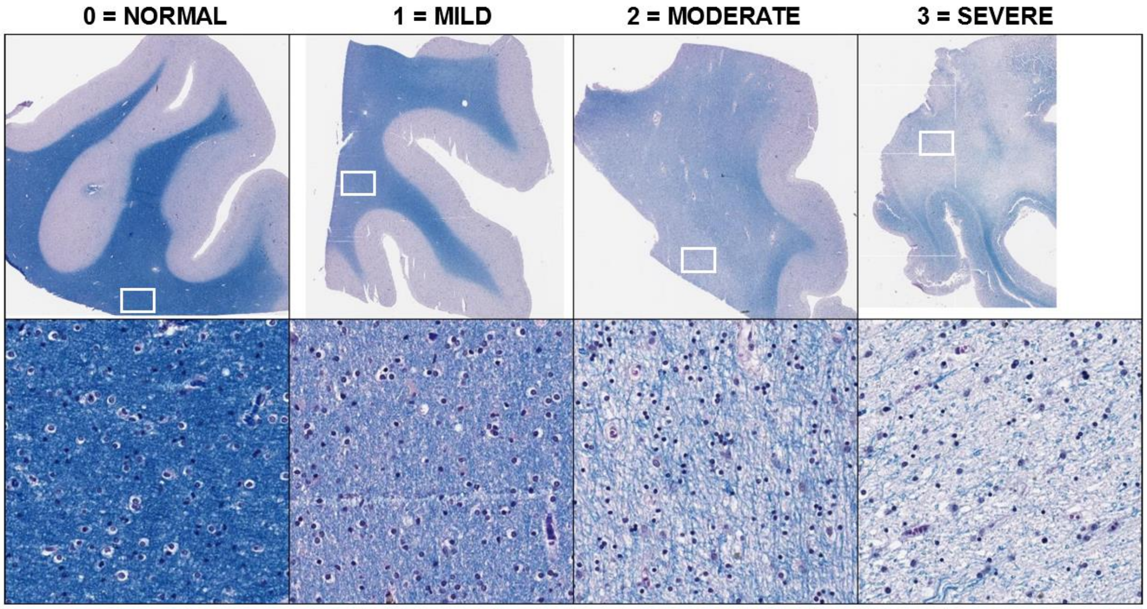

Exemplar photomicrographs of ordinal rating system for luxol fast blue (LFB) myelin staining of WM neurodegeneration depicting normal healthy myelin with confluent LFB staining and highly organized fibers (score = 0), mild reduced LFB stain and disorganization of fibers (score = 1), moderately reduced LFB and increased disorganization of fibers (score = 2) and nearly absent LFB staining and extremely disorganized WM fibers (score = 3). Upper row is 1x magnification view, white box depicts area of 32x view displayed below in bottom row.

Supplementary Figure 3. Association of WM %AO measurements with LFB ordinal ratings of WM degeneration

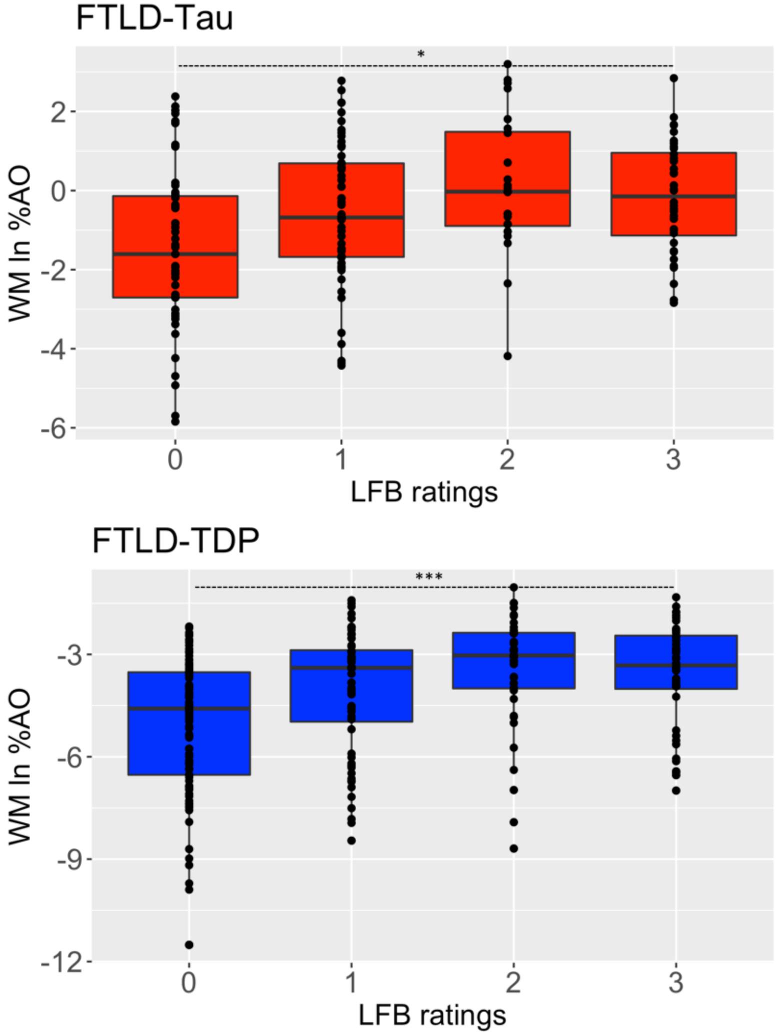

Plots portray the relationship between ordinal ratings of WM degeneration based on LFB stain and digitally acquired %AO scores of WM pathology after natural logarithmic transformation in FTLD-Tau (above) and FTLD-TDP (below). We found a significant positive association in both FTLD-Tau (beta = 0.32, SE = 0.11, $p = 0.002$) and FTLD-TDP (beta = 0.40, SE = 0.09, $p < 0.001$).

Legend: %AO = percentage area occupied by pathology; FTLD-Tau = frontotemporal lobar degeneration with inclusions of the tau protein; FTLD-TDP = frontotemporal lobar degeneration with inclusions of the TDP-43 protein; LFB = luxol fast blue; WM = white matter.

Supplementary Table 1. Empirically derived stain detection algorithms for tau and TDP-43 pathology in each staining batch

	Hematoxylin (RGB)			DAB (RGB)			Min. OD
**Tau Batch 1**	0.327	0.369	0.146	0.452	0.716	0.666	0.185
**Tau Batch 2**	0.248	0.276	0.108	0.503	0.763	0.716	0.217
**Tau Batch 3**	0.296	0.331	0.121	0.487	0.621	0.493	0.183
**Tau Batch 4**	0.219	0.258	0.0946	0.466	0.627	0.547	0.192
**TDP-43 Batch 1**	0.2634	0.2784	0.0962	0.3938	0.5382	0.4942	0.246
**TDP-43 Batch 2**	0.240	0.2692	0.092	0.3636	0.531	0.4854	0.208
**TDP-43 Batch 3**	0.183	0.1976	0.072	0.2882	0.3818	0.33	0.191
**TDP-43 Batch 4**	0.2452	0.2708	0.093	0.3516	0.5152	0.501	0.217

Legend: Batch = staining batch; RGB = Red, Green and Blue color deconvolution settings; Stain Min. OD = minimum OD value of DAB (Diaminobenzine) chromogen to differentiate pathology from background stain for % area occupied (%AO) calculation (% DAB positive pixels / total tissue pixels per ROI).

Supplementary Table 2. Availability of GM and WM AO% measurements in core and extended regions by proteinopathy groups and subtypes

	Core					Extended			
**Left-hemi GM**	**ACG**	**ANG**	**MFC**	**OFC**	**STG**	**INS**	**SPL**	**VLT**	**Tot GM**
FTLD-Tau	21	21	21	21	18	13	15	13	143
CBD	7	6	7	8	5	7	7	6	53
MAPT	1	1	1	2	1	1	1	1	14
PiD	7	8	7	5	6	2	4	4	38
PSP	6	6	6	6	6	3	3	2	38
FTLD-TDP	34	29	31	39	30	12	14	12	201
Type A	13	12	12	15	13	8	9	9	91
Type B	11	8	9	12	7	1	1	1	50
Type C	7	7	8	9	8	2	2	2	45
Type E	3	2	2	3	2	1	2	0	15
All	55	50	52	60	48	25	29	25	344
**Left-hemi WM**	**ACG**	**ANG**	**MFC**	**OFC**	**STG**	**INS**	**SPL**	**VLT**	**Tot WM**
FTLD-Tau	21	19	21	23	17	13	15	12	141
CBD	7	6	7	8	4	7	7	5	51
MAPT	1	1	1	2	1	1	1	1	14
PiD	7	7	7	7	6	2	4	5	40
PSP	6	5	6	6	6	3	3	1	36
FTLD-TDP	35	29	31	40	30	12	14	12	203
Type A	13	12	13	15	13	7	9	9	91
Type B	11	8	8	12	7	1	1	1	49
Type C	8	7	8	10	8	2	2	2	47
Type E	3	2	2	3	2	2	2	0	16
All	56	48	52	63	47	25	29	24	344
**Right-hemi GM**	**ACG**	**ANG**	**MFC**	**OFC**	**STG**	**INS**	**SPL**	**VLT**	**Tot GM**
FTLD-Tau	19	16	18	22	18	12	15	9	129
CBD	5	5	6	7	5	6	7	5	46
MAPT	4	4	4	4	4	1	1	1	23
PiD	5	4	3	6	5	2	4	2	31
PSP	5	3	5	5	4	3	3	1	29
FTLD-TDP	27	25	26	31	23	11	13	9	165
Type A	11	8	10	14	8	6	8	6	71
Type B	7	8	8	8	7	1	1	1	41
Type C	6	6	5	6	5	2	2	2	34
Type E	3	3	3	3	3	2	2	0	19
All	46	41	44	53	41	23	28	18	294

Right-hemi WM	ACG	ANG	MFC	OFC	STG	INS	SPL	VLT	Tot WM
FTLD-Tau	21	15	19	23	19	10	15	11	133
CBD	5	5	6	7	5	6	7	5	46
MAPT	4	3	4	4	4	0	1	1	21
PiD	7	4	4	7	6	3	4	3	38
PSP	5	3	5	5	4	1	3	2	28
FTLD-TDP	27	26	26	31	23	12	14	10	169
Type A	11	9	10	14	8	7	9	7	75
Type B	7	8	8	8	7	1	1	1	41
Type C	6	6	5	6	5	2	2	2	34
Type E	3	3	3	3	3	2	2	0	19
All	48	41	45	54	42	22	29	21	302

Legend: ACG = anterior cingulate gyrus; ANG = angular gyrus; CBD = corticobasal degeneration; FTLD-Tau = frontotemporal lobar degeneration with inclusions of the tau protein; FTLD-TDP = frontotemporal lobar degeneration with inclusions of the TDP-43 protein; GM = grey matter; hemi = hemisphere; INS = anterior insular cortex; L = left; MAPT = FTLD-tau with *MAPT* gene mutation; MFC = mid-frontal cortex; OFC = orbitofrontal cortex; PiD = Pick’s disease; PSP = progressive supranuclear palsy; R = right; STG = superior temporal gyrus; SPL = superior parietal lobule; Type A/Type B/Type C/Type E = subtypes of FTLD-TDP proteinopathy; VLT = ventrolateral temporal cortex; WM = white matter.

Table displays the availability of digital pathology data per region in grey and white matter of both hemispheres in proteinopathy groups (FTLD-Tau vs. FTLD-TDP) and their subtypes. We distinguish “core” regions (i.e. ACG, ANG, MFC, OFC, STG), which were consistently sampled at autopsy in all patients according to standard neuropathological sampling procedures with random-hemisphere sampling (Toledo *et al.*, 2014), and “extended” regions (i.e. INS, SPL, VLT), which were sampled only more recently (from 2005 onwards) in a subset of cases (FTLD-Tau = 16, FTLD-TDP = 14) bilaterally. Because of retrospective data acquisition, we excluded missing data and damaged tissues.

Supplementary Table 3. Comparison of WM pathology burden between FTLD-Tau and FTLD-TDP

**Absolute WM pathology in FTLD-Tau vs. FTLD-TDP**			
Dependent: ln WM %AO			
Fixed effects:	*estimate*	*SE*	*p-value*
(Intercept)	-0.28	0.41	0.488
**Proteinopathy [FTLD-Tau]**	**4.21**	**0.34**	**<0.001**
Region [ANG]	-0.27	0.18	0.124
Region [INS]	0.33	0.23	0.141
Region [MFC]	0.26	0.17	0.128
Region [OFC]	0	0.17	1.000
Region [SPL]	-0.76	0.21	<0.001
Region [STG]	0.21	0.18	0.249
Region [VLT]	0.11	0.23	0.629
Hemisphere [R]	-0.11	0.13	0.397
Mutation status	1.39	0.35	<0.001
Disease duration	-0.07	0.04	0.051

Legend: %AO = percentage area occupied by pathology; ANG = angular gyrus; FTLD-Tau = frontotemporal lobar degeneration with inclusions of the tau protein; FTLD-TDP = frontotemporal lobar degeneration with inclusions of the TDP-43 protein; INS = anterior insular cortex; MFC = mid-frontal cortex; OFC = orbitofrontal cortex; SE = standard error; STG = superior temporal gyrus; SPL = superior parietal lobule; VLT = ventrolateral temporal cortex; WM = white matter.

Table displays a LME model in the total cohort using ln-transformed WM %AO as dependent variable, proteinopathy group as fixed-effect predictor of interest (highlighted in bold), and region, hemisphere, mutation status and disease duration as fixed-effects covariates.

Supplementary Table 4. Comparison of WM pathology burden between all proteinopathy subtypes

**Absolute WM pathology between proteinopathy subtypes**			
Dependent: ln WM %AO			
Fixed effects:	*df*	*F-value*	*p-value*
**Subtype**	**7,83**	**60.2**	**0.000**
Region	7,559	5.4	0.000
Hemisphere	1,616	1.4	0.238
Mutation status	1,87	9.1	0.003
Disease duration	1,83	0.0	0.864
***Post-hoc* comparisons**			
Dependent: ln WM %AO			
Pairwise contrasts:	*estimate*	*SE*	*p-value*
CBD - MAPT	1.79	0.65	0.122
CBD - PiD	2.36	0.44	0.000
CBD - PSP	3.23	0.45	0.000
CBD - Type A	5.36	0.45	0.000
CBD - Type B	5.41	0.47	0.000
CBD - Type C	7.64	0.44	0.000
CBD - Type E	4.39	0.56	0.000
MAPT - PiD	0.56	0.65	0.988
MAPT - PSP	1.43	0.67	0.400
MAPT - Type A	3.57	0.53	0.000
MAPT - Type B	3.62	0.54	0.000
MAPT - Type C	5.85	0.62	0.000
MAPT - Type E	2.60	0.75	0.019
PiD - PSP	0.87	0.47	0.589
PiD - Type A	3.01	0.45	0.000
PiD - Type B	3.06	0.48	0.000
PiD - Type C	5.29	0.42	0.000
PiD - Type E	2.03	0.60	0.022
PSP - Type A	2.14	0.47	0.001
PSP - Type B	2.19	0.49	0.001
PSP - Type C	4.42	0.46	0.000
PSP - Type E	1.16	0.58	0.482
Type A - Type B	0.05	0.34	1.000
Type A - Type C	2.28	0.42	0.000
Type A - Type E	-0.97	0.58	0.710
Type B - Type C	2.23	0.44	0.000
Type B - Type E	-1.02	0.59	0.670
Type C - Type E	-3.25	0.59	0.000

Legend: %AO = percentage area occupied by pathology; CBD = corticobasal degeneration; MAPT = FTLD-tau with *MAPT* gene mutation; PiD = Pick's disease; PSP = progressive supranuclear palsy; SE = standard error; Type A/Type B/Type C/Type E = subtypes of FTLD-TDP proteinopathy; WM = white matter.

Table displays a LME-based ANOVA in the total cohort using ln-transformed WM %AO as dependent variable, proteinopathy subtype as fixed-effect predictor of interest (highlighted in bold), and region, hemisphere, mutation status and disease duration as fixed-effect covariates. For this significant analysis, we also include a planned *post-hoc* analysis with pairwise comparisons between all proteinopathy subtypes with Tukey correction for multiple comparisons.

Supplementary Table 5. Comparison of WM-to-GM ratio between FTLD-Tau and FTLD-TDP

**WM-to-GM ratio in FTLD-Tau vs. FTLD-TDP**			
Dependent: ln WM-to-GM ratio			
Fixed effects:	*estimate*	*SE*	*p-value*
(Intercept)	-0.46	0.39	0.246
**Proteinopathy [FTLD-Tau]**	**2.09**	**0.33**	**<0.001**
Region [ANG]	0.45	0.18	0.01
Region [INS]	0.82	0.23	<0.001
Region [MFC]	0.70	0.17	<0.001
Region [OFC]	0.27	0.17	0.102
Region [SPL]	0.52	0.21	0.013
Region [STG]	0.86	0.18	<0.001
Region [VLT]	0.26	0.23	0.269
Hemisphere [R]	0.09	0.13	0.454
Mutation Status	1.19	0.34	<0.001
Disease Duration	-0.13	0.04	<0.001

Legend: ANG = angular gyrus; FTLD-Tau = frontotemporal lobar degeneration with inclusions of the tau protein; FTLD-TDP = frontotemporal lobar degeneration with inclusions of the TDP-43 protein; GM = grey matter; INS = anterior insular cortex; MFC = mid-frontal cortex; OFC = orbitofrontal cortex; SE = standard error; STG = superior temporal gyrus; SPL = superior parietal lobule; VLT = ventrolateral temporal cortex; WM = white matter.

Table displays a LME model in the total cohort using ln-transformed WM-to-GM ratio as dependent variable, proteinopathy group as fixed-effect predictor of interest (highlighted in bold), and region, hemisphere, mutation status and disease duration as fixed-effect covariates.

Supplementary Table 6. Comparison of WM-to-GM ratio between proteinopathy subtypes

**WM-to-GM ratio between proteinopathy subtypes**			
Dependent: ln WM-to-GM ratio			
Fixed effects:	*df*	*F-value*	*p-value*
**Subtype**	**7,84**	**32.3**	**0.000**
Region	7,544	5.2	0.000
Hemisphere	1,579	0.3	0.559
Mutation status	1,88	4.9	0.029
Disease duration	1,84	0.1	0.737
***Post-hoc* comparisons**			
Dependent: ln WM-to-GM ratio			
Pairwise contrasts:	*estimate*	*SE*	*p-value*
CBD - MAPT	1.33	0.58	0.312
CBD - PiD	2.18	0.40	0.000
CBD - PSP	1.16	0.40	0.097
CBD - Type A	2.51	0.40	0.000
CBD - Type B	2.09	0.42	0.000
CBD - Type C	5.47	0.39	0.000
CBD - Type E	1.28	0.50	0.182
MAPT - PiD	0.85	0.58	0.827
MAPT - PSP	-0.18	0.60	1.000
MAPT - Type A	1.17	0.47	0.211
MAPT - Type B	0.76	0.48	0.768
MAPT - Type C	4.13	0.56	0.000
MAPT - Type E	-0.05	0.67	1.000
PiD - PSP	-1.03	0.42	0.239
PiD - Type A	0.32	0.40	0.993
PiD - Type B	-0.10	0.43	1.000
PiD - Type C	3.28	0.38	0.000
PiD - Type E	-0.90	0.53	0.692
PSP - Type A	1.35	0.42	0.041
PSP - Type B	0.93	0.44	0.408
PSP - Type C	4.31	0.41	0.000
PSP - Type E	0.13	0.52	1.000
Type A - Type B	-0.42	0.31	0.873
Type A - Type C	2.96	0.37	0.000
Type A - Type E	-1.22	0.52	0.281
Type B - Type C	3.38	0.40	0.000
Type B - Type E	-0.81	0.53	0.790
Type C - Type E	-4.19	0.53	0.000

Legend: CBD = corticobasal degeneration; GM = grey matter; MAPT = FTLD-tau with *MAPT* gene mutation; PiD = Pick’s disease; PSP = progressive supranuclear palsy; SE = standard error; Type A/Type B/Type C/Type E = subtypes of FTLD-TDP proteinopathy; WM = white matter.

Table displays a LME model in the total cohort using ln-transformed WM-to-GM ratio as dependent variable, proteinopathy subtype as fixed-effect predictor of interest (highlighted in bold), and region, hemisphere, mutation status and disease duration as fixed-effect covariates. Pairwise comparisons

between proteinopathy subtypes were performed using a *post-hoc* analysis on LME-derived least-square means with Tukey correction for multiple comparisons.

Supplementary Table 7. Comparison of WM pathology burden between genetic subtypes

**(A) Genetic vs. sporadic cases across pathologies**			
Dependent: ln WM %AO			
Fixed effects:	*estimate*	*SE*	*p-value*
(Intercept)	0.97	0.38	0.010
**Mutation status**	**1.02**	**0.34**	**0.003**
Hemisphere [R]	-0.14	0.12	0.237
Region [ANG]	-0.27	0.18	0.123
Region [INS]	0.29	0.23	0.198
Region [MFC]	0.26	0.17	0.133
Region [OFC]	0	0.17	0.982
Region [SPL]	-0.8	0.21	<0.001
Region [STG]	0.22	0.18	0.222
Region [VLT]	0.08	0.23	0.723
Subtypes [MAPT]	-1.79	0.65	0.006
Subtypes [PiD]	-2.36	0.44	<0.001
Subtypes [PSP]	-3.23	0.45	<0.001
Subtypes [Type A]	-5.36	0.45	<0.001
Subtypes [Type B]	-5.41	0.47	<0.001
Subtypes [Type C]	-7.64	0.44	<0.001
Subtypes [Type E]	-4.39	0.56	<0.001
Disease duration	0.01	0.03	0.864
**(B) Specific genetic mutations in FTLD-TDP**			
Dependent: ln WM %AO			
Fixed effects:	*df*	*F-value*	*p-value*
**Mutation**	**3,49**	**6.2**	**0.001**
Hemisphere	1,357	1.5	0.229
Region	7,312	1.9	0.062
Disease duration	1,49	7.6	0.008
***Post-hoc* comparisons**			
Dependent: ln WM %AO			
Pairwise contrasts:	*estimate*	*SE*	*p-value*
None - C9orf72	-1.28	0.45	0.031
None - GRN	-1.96	0.50	0.001
None - TBK1	-0.37	1.03	0.984
C9orf72 - GRN	-0.68	0.55	0.604
C9orf72 - TBK1	0.91	1.05	0.826
GRN - TBK1	1.58	1.07	0.460
**(C) Specific genetic mutations in FTLD-Tau**			
Dependent: ln WM %AO			
Fixed effects:	*df*	*F-value*	*p-value*
**Mutation**	**1,36**	**1.5**	**0.230**
Hemisphere	1,257	0.0	0.962
Region	7,232	4.6	0.000
Disease duration	1,34	0.0	0.910

Legend: %AO = percentage area occupied by pathology; ANG = angular gyrus; C9orf72 = FTLD-TDP with *C9orf72* gene mutation; FTLD-Tau = frontotemporal lobar degeneration with inclusions of the tau protein; FTLD-TDP = frontotemporal lobar degeneration with inclusions of the TDP-43 protein; GRN = FTLD-TDP with *GRN* gene mutation; INS = anterior insular cortex; MAPT = FTLD-tau with *MAPT* gene mutation; MFC = mid-frontal cortex; OFC = orbitofrontal cortex; PiD = Pick's disease; PSP = progressive supranuclear palsy; SE = standard error; STG = superior temporal gyrus; SPL = superior parietal lobule; Type A/Type B/Type C/Type E = subtypes of FTLD-TDP proteinopathy; VLT = ventrolateral temporal cortex; WM = white matter.

Section (A) displays a LME model in the total cohort using ln-transformed WM %AO as dependent variable, mutation status (genetic vs. sporadic) as fixed-effect predictor of interest (highlighted in bold), and region, hemisphere, proteinopathy subtype and disease duration as fixed-effect covariates.
Section (B) displays a LME-based ANOVA in FTLD-TDP using ln-transformed WM %AO as dependent variable, FTLD-TDP-related mutations (*C9orf72* vs. *GRN* vs. *TBK1* vs. sporadic) as fixed-effect predictor of interest (highlighted in bold), and region, hemisphere and disease duration as fixed-effect covariates. For this significant analysis, we also include a planned *post-hoc* analysis with pairwise comparisons between specific FTLD-TDP-related mutations and sporadic cases with Tukey correction for multiple comparisons.
Section (C) displays a LME-based ANOVA in FTLD-Tau using ln-transformed WM %AO as dependent variable, FTLD-Tau-related mutation (*MAPT* vs. sporadic) as fixed-effect predictor of interest (highlighted in bold), and region, hemisphere and disease duration as fixed-effect covariates.

Supplementary Table 8. Comparison of LFB ordinal ratings between FTLD-Tau and FTLD-TDP overall

**WM degeneration between proteinopathies**			
Dependent: LFB ordinal ratings			
Fixed effects:	*estimate*	*SE*	*p-value*
(Intercept)	0.89	0.23	<0.001
**Proteinopathy [FTLD-Tau]**	**0.36**	**0.18**	**0.047**
Region [ANG]	-0.29	0.13	0.027
Region [MFC]	0.02	0.13	0.900
Region [OFC]	0.15	0.13	0.266
Region [STG]	0.21	0.13	0.113
Hemisphere [R]	0.00	0.13	0.972
Mutation status	0.38	0.18	0.037
Disease duration	0.04	0.02	0.031
**Interaction between proteinopathy group and region (LM E-based ANOVA)**			
Dependent: LFB ordinal ratings			
Fixed effects:	*df*	*F-value*	*p-value*
**Proteinopathy:Region**	**4,341**	**3.9**	**0.004**
Proteinopathy	1,85	3.6	0.061
Region	4,342	3.8	0.005
Hemisphere	1,266	0.0	0.990
Mutation status	1,85	4.1	0.045
Disease duration	1,87	4.9	0.030

Legend: ANG = angular gyrus; FTLD-Tau = frontotemporal lobar degeneration with inclusions of the tau protein; FTLD-TDP = frontotemporal lobar degeneration with inclusions of the TDP-43 protein; LFB = luxol fast blue; MFC = mid-frontal cortex; OFC = orbitofrontal cortex; SE = standard error; STG = superior temporal gyrus; WM = white matter.

Section (A) displays a LME model using LFB ordinal ratings as dependent variable, proteinopathy group as fixed-effect predictor of interest (highlighted in bold), and region, hemisphere, mutation status and disease duration as fixed-effect covariates.
Section (B) displays a LME-based ANOVA to test the interaction between proteinopathy group and region on LFB ordinal ratings as dependent variable. The model used the same variables as section A, with the addition of the interaction term(highlighted in bold).

Supplementary Table 9. Association of WM pathology burden with LFB ordinal ratings in FTLD-Tau and FTLD-TDP

**(A) Association in FTLD-Tau**			
Dependent: ln WM %AO			
Fixed effects:	*estimate*	*SE*	*p-value*
(Intercept)	0.39	0.45	0.382
**LFB ordinal ratings**	**0.32**	**0.10**	**0.002**
Region [ANG]	-0.76	0.27	0.006
Region [MFC]	-0.06	0.26	0.809
Region [OFC]	-0.6	0.27	0.024
Region [STG]	-0.24	0.27	0.369
Hemisphere [R]	0.08	0.25	0.744
Subtypes [MAPT]	-1.01	0.47	0.03
Subtypes [PiD]	-2.74	0.41	<0.001
Subtypes [PSP]	-2.91	0.39	<0.001
Disease duration	0.08	0.05	0.102
**(B) Association in FTLD-TDP**			
Dependent: ln WM %AO			
Fixed effects:	*estimate*	*SE*	*p-value*
(Intercept)	-4.35	0.48	<0.001
**LFB ordinal ratings**	**0.40**	**0.08**	**<0.001**
Region [ANG]	0.11	0.22	0.617
Region [MFC]	0.20	0.22	0.373
Region [OFC]	-0.15	0.22	0.492
Region [STG]	0.15	0.23	0.505
Hemisphere [R]	-0.55	0.22	0.013
Subtypes [Type B]	0.30	0.34	0.384
Subtypes [Type C]	-2.24	0.41	<0.001
Subtypes [Type E]	0.89	0.57	0.120
Mutation status	0.79	0.33	0.016
Disease duration	-0.04	0.04	0.268

Legend: %AO = percentage area occupied by pathology; ANG = angular gyrus; FTLD-Tau = frontotemporal lobar degeneration with inclusions of the tau protein; FTLD-TDP = frontotemporal lobar degeneration with inclusions of the TDP-43 protein; LFB = luxol fast blue; MAPT = FTLD-tau with *MAPT* gene mutation; MFC = mid-frontal cortex; OFC = orbitofrontal cortex; PiD = Pick's disease; PSP = progressive supranuclear palsy; SE = standard error; STG = superior temporal gyrus; Type B/Type C/Type E = subtypes of FTLD-TDP proteinopathy; WM = white matter.

Section (A) displays a LME model in FTLD-Tau using ln-transformed WM %AO as dependent variable, LFB ordinal ratings as fixed-effect predictor of interest (highlighted in bold), and region, hemisphere, proteinopathy subtype and disease duration as fixed-effect covariates.
Section (B) displays a LME model in FTLD-TDP using ln-transformed WM %AO as dependent variable, LFB ordinal ratings as fixed-effect predictor of interest (highlighted in bold), and region, hemisphere, proteinopathy subtype, mutation status and disease duration as fixed-effect covariates.

Supplementary Table 10. Regional distribution of GM and WM pathology burden in FTLD-Tau

**FTLD-Tau GM**				**FTLD-Tau WM**			
Dependent: ln GM %AO				*Dependent: ln WM %AO*			
Fixed effects:	*df*	*F*	*p*	*Fixed effects:*	*df*	*F*	*p*
**Region**	**4,156**	**7.0**	**0.000**	**Region**	**4,158**	**3.4**	**0.010**
Hemisphere	1,170	0.7	0.409	Hemisphere	1,146	1.2	0.284
Subtype	3,32	6.3	0.002	Subtype	3,32	26.9	0.000
Disease duration	1,32	1.3	0.263	Disease duration	1,31	2.6	0.117
**Least-square means**				**Least-square means**			
Region	*est*	*SE*		*Region*	*est*	*SE*	
ACG	0.77	0.27		ACG	-0.42	0.22	
ANG	-0.24	0.28		ANG	-1.15	0.24	
MFC	0.47	0.27		MFC	-0.21	0.23	
OFC	-0.29	0.27		OFC	-0.70	0.22	
STG	-0.42	0.28		STG	-0.46	0.24	
***Post-hoc* comparisons**				***Post-hoc* comparisons**			
Dependent: ln GM %AO				*Dependent: ln WM %AO*			
Pairwise contrasts:	*est*	*SE*	*p*	*Pairwise contrasts:*	*est*	*SE*	*p*
ACG - ANG	1.01	0.28	0.005	ACG - ANG	0.73	0.27	0.057
ACG - MFC	0.30	0.28	0.819	ACG - MFC	-0.21	0.26	0.926
ACG - OFC	1.06	0.27	0.002	ACG - OFC	0.28	0.25	0.793
ACG - STG	1.19	0.29	0.001	ACG - STG	0.03	0.27	1.000
ANG - MFC	-0.71	0.29	0.100	ANG - MFC	-0.94	0.27	0.006
ANG - OFC	0.05	0.28	1.000	ANG - OFC	-0.45	0.27	0.443
ANG - STG	0.19	0.29	0.969	ANG - STG	-0.70	0.28	0.102
MFC - OFC	0.76	0.27	0.050	MFC - OFC	0.49	0.25	0.300
MFC - STG	0.89	0.29	0.020	MFC - STG	0.25	0.27	0.893
OFC - STG	0.13	0.28	0.990	OFC - STG	-0.25	0.26	0.879

Legend: %AO = percentage area occupied by pathology; ACG = anterior cingulate gyrus; ANG = angular gyrus; est = estimate; F = F-value; FTLD-Tau = frontotemporal lobar degeneration with inclusions of the tau protein; GM = grey matter; MFC = mid-frontal cortex; OFC = orbitofrontal cortex; p = p-value; SE = standard error; STG = superior temporal gyrus; WM = white matter.

Table displays two LME-based ANOVAs in FTLD-Tau using ln-transformed GM or WM %AO as dependent variables, region as fixed-effect predictor of interest (highlighted in bold), and hemisphere, proteinopathy subtype and disease duration as fixed-effect covariates. For both significant analyses in GM and WM, we also include a planned *post-hoc* analysis with pairwise comparisons between all regions with Tukey correction for multiple comparisons.

Supplementary Table 11. Regional distribution of GM and WM pathology burden in FTLD-TDP

**FTLD-TDP GM**				**FTLD-TDP WM**			
Dependent: ln GM %AO				*Dependent: ln WM %AO*			
Fixed effects:	*df*	*F*	*p*	*Fixed effects:*	*df*	*F*	*p*
**Region**	**4,240**	**8.2**	**0.000**	**Region**	**4,239**	**1.2**	**0.297**
Hemisphere	1,232	3.1	0.077	Hemisphere	1,272	0.6	0.421
Subtype	3,50	5.1	0.004	Subtype	3,47	11.3	0.000
Mutation status	1,51	2.1	0.150	Mutation status	1,48	8.4	0.006
Disease duration	1,50	0.1	0.811	Disease duration	1,48	0.8	0.382
**Least-square means**				**Least-square means**			
Region	*est*	*SE*		*Region*	*est*	*SE*	
ACG	-1.71	0.17		ACG	-4.43	0.23	
ANG	-2.37	0.17		ANG	-4.39	0.23	
MFC	-2.29	0.17		MFC	-4.13	0.23	
OFC	-1.56	0.16		OFC	-4.23	0.22	
STG	-2.12	0.17		STG	-4.04	0.23	
***Post-hoc* comparisons**							
Dependent: ln GM %AO							
Pairwise contrasts:	*est*	*SE*	*p*				
ACG - ANG	0.66	0.18	0.003				
ACG - MFC	0.58	0.18	0.012				
ACG - OFC	-0.15	0.17	0.912				
ACG - STG	0.41	0.18	0.172				
ANG - MFC	-0.08	0.18	0.993				
ANG - OFC	-0.80	0.18	0.000				
ANG - STG	-0.25	0.19	0.670				
MFC - OFC	-0.72	0.17	0.000				
MFC - STG	-0.17	0.19	0.887				
OFC - STG	0.55	0.18	0.017				

Legend: %AO = percentage area occupied by pathology; ACG = anterior cingulate gyrus; ANG = angular gyrus; est = estimate; F = F-value; FTLD-TDP = frontotemporal lobar degeneration with inclusions of the TDP-43 protein; GM = grey matter; MFC = mid-frontal cortex; OFC = orbitofrontal cortex; p = p-value; SE = standard error; STG = superior temporal gyrus; WM = white matter.

Table displays two LME-based ANOVAs in FTLD-TDP using ln-transformed GM or WM %AO as dependent variables, region as fixed-effect predictor of interest (highlighted in bold), and hemisphere, proteinopathy subtype, mutation status and disease duration as fixed-effect covariates. For the significant analysis in GM, we also include a planned *post-hoc* analysis with pairwise comparisons between all regions with Tukey correction for multiple comparisons.

Supplementary Table 12. Sub-analysis: regional distribution of GM and WM pathology burden in FTLD-Tau subtypes

**CBD GM**				**CBD WM**			
Dependent: ln GM %AO				*Dependent: ln WM %AO*			
Fixed effects:	*df*	*F*	*p*	*Fixed effects:*	*df*	*F*	*p*
**Region**	**4,45**	**3.8**	**0.009**	**Region**	**4,44**	**2.3**	**0.075**
Hemisphere	1,54	0.0	0.861	Hemisphere	1,51	0.0	0.834
Disease duration	1,9	0.3	0.574	Disease duration	1,9	0.1	0.791
**Least-square means**				**Least-square means**			
Region	*est*	*SE*		*Region*	*est*	*SE*	
ACG	0.98	0.40		ACG	1.09	0.40	
ANG	0.65	0.41		ANG	0.48	0.40	
MFC	1.33	0.40		MFC	1.39	0.39	
OFC	0.19	0.39		OFC	1.02	0.39	
STG	0.10	0.42		STG	0.94	0.42	
**PSP GM**				**PSP WM**			
Dependent: ln GM %AO				*Dependent: ln WM %AO*			
Fixed effects:	*df*	*F*	*p*	*Fixed effects:*	*df*	*F*	*p*
**Region**	**4,38**	**3.4**	**0.019**	**Region**	**4,37**	**3.3**	**0.021**
Hemisphere	1,44	0.2	0.645	Hemisphere	1,17	0.4	0.525
Disease duration	1,7	0.5	0.520	Disease duration	1,4	0.7	0.436
**Least-square means**				**Least-square means**			
Region	*est*	*SE*		*Region*	*est*	*SE*	
ACG	-0.91	0.52		ACG	-1.92	0.36	
ANG	-1.07	0.55		ANG	-1.77	0.42	
MFC	-1.24	0.52		MFC	-1.98	0.36	
OFC	-2.34	0.52		OFC	-3.30	0.36	
STG	-1.96	0.53		STG	-2.26	0.37	
**PiD GM**				**PiD WM**			
Dependent: ln GM %AO				*Dependent: ln WM %AO*			
Fixed effects:	*df*	*F*	*p*	*Fixed effects:*	*df*	*F*	*p*
**Region**	**4,42**	**5.3**	**0.002**	**Region**	**4,47**	**3.1**	**0.023**
Hemisphere	1,38	2.0	0.169	Hemisphere	1,30	0.9	0.360
Disease duration	1,9	0.5	0.503	Disease duration	1,8	1.7	0.225
**Least-square means**				**Least-square means**			
Region	*est*	*SE*		*Region*	*est*	*SE*	
ACG	2.36	0.54		ACG	-1.04	0.42	
ANG	-0.28	0.54		ANG	-2.61	0.47	
MFC	1.17	0.58		MFC	-0.78	0.47	
OFC	0.66	0.56		OFC	-0.77	0.42	
STG	0.00	0.56		STG	-0.91	0.45	
**MAPT GM**				**MAPT WM**			
Dependent: ln GM %AO				*Dependent: ln WM %AO*			
Fixed effects:	*df*	*F*	*p*	*Fixed effects:*	*df*	*F*	*p*

**Region**	**4,17**	**0.3**	**0.853**	**Region**	**4,18**	**1.1**	**0.392**
Hemisphere	1,12	1.0	0.328	Hemisphere	1,18	10.8	0.004
Disease duration	1,3	3.6	0.159	Disease duration	1,18	12.8	0.002
**Least-square means**				**Least-square means**			
Region	*est*	*SE*		*Region*	*est*	*SE*	
ACG	0.56	0.71		ACG	0.34	0.41	
ANG	0.25	0.71		ANG	0.06	0.45	
MFC	0.93	0.71		MFC	1.04	0.41	
OFC	0.68	0.64		OFC	0.19	0.36	
STG	0.62	0.71		STG	0.72	0.41	

Legend: %AO = percentage area occupied by pathology; ANG = angular gyrus; CBD = corticobasal degeneration; est = estimate; F = f-value; FTLD-Tau = frontotemporal lobar degeneration with inclusions of the tau protein; MAPT = FTLD-tau with *MAPT* gene mutation; MFC = mid-frontal cortex; OFC = orbitofrontal cortex; PiD = Pick's disease; PSP = progressive supranuclear palsy; SE = standard error; STG = superior temporal gyrus; WM = white matter.

Supplementary Table 13. Sub-analysis: regional distribution of GM and WM pathology burden in FTLD-TDP subtypes

**Type A GM**				**Type A WM**			
Dependent: ln GM %AO				*Dependent: ln WM %AO*			
Fixed effects:	*df*	*F*	*p*	*Fixed effects:*	*df*	*F*	*p*
**Region**	**4,94**	**3.79**	**0.007**	**Region**	**4,92**	**0.40**	**0.810**
Hemisphere	1,99	3.60	0.061	Hemisphere	1,111	1.56	0.215
Disease duration	1,20	0.01	0.937	Disease duration	1,16	0.80	0.383
**Least-square means**				**Least-square means**			
Region	*est*	*SE*		*Region*	*est*	*SE*	
ACG	-1.70	0.25		ACG	-3.85	0.38	
ANG	-2.18	0.27		ANG	-3.66	0.39	
MFC	-2.31	0.26		MFC	-3.42	0.39	
OFC	-1.34	0.24		OFC	-3.70	0.37	
STG	-2.11	0.27		STG	-3.61	0.39	
**Type B GM**				**Type B WM**			
Dependent: ln GM %AO				*Dependent: ln WM %AO*			
Fixed effects:	*df*	*F*	*p*	*Fixed effects:*	*df*	*F*	*p*
**Region**	**4,62**	**3.54**	**0.011**	**Region**	**4,61**	**2.55**	**0.048**
Hemisphere	1,55	0.47	0.494	Hemisphere	1,76	1.15	0.286
Disease duration	1,12	0.15	0.704	Disease duration	1,13	0.22	0.649
**Least-square means**				**Least-square means**			
Region	*est*	*SE*		*Region*	*est*	*SE*	
ACG	-2.21	0.31		ACG	-3.96	0.30	
ANG	-3.12	0.32		ANG	-3.80	0.31	
MFC	-2.25	0.32		MFC	-3.22	0.31	
OFC	-1.93	0.30		OFC	-3.59	0.30	
STG	-2.31	0.34		STG	-3.80	0.32	
**Type C GM**				**Type C WM**			
Dependent: ln GM %AO				*Dependent: ln WM %AO*			
Fixed effects:	*df*	*F*	*p*	*Fixed effects:*	*df*	*F*	*p*
**Region**	**4,51**	**8.44**	**0.000**	**Region**	**4,53**	**2.22**	**0.080**
Hemisphere	1,48	0.00	0.978	Hemisphere	1,45	1.43	0.238
Disease duration	1,10	2.89	0.120	Disease duration	1,10	3.09	0.109
**Least-square means**				**Least-square means**			
Region	*est*	*SE*		*Region*	*est*	*SE*	
ACG	-0.96	0.23		ACG	-6.90	0.47	
ANG	-1.78	0.22		ANG	-7.07	0.48	
MFC	-1.99	0.23		MFC	-6.75	0.50	
OFC	-0.82	0.22		OFC	-6.58	0.46	
STG	-1.20	0.23		STG	-5.57	0.49	
**Type E GM**				**Type E WM**			
Dependent: ln GM %AO				*Dependent: ln WM %AO*			
Fixed effects:	*df*	*F*	*p*	*Fixed effects:*	*df*	*F*	*p*

**Region**	**4,18**	**2.31**	**0.098**	**Region**	**4,20**	**1.55**	**0.227**
Hemisphere	1,11	0.07	0.800	Hemisphere	1,20	0.16	0.697
Disease duration	1,3	0.62	0.498	Disease duration	1,20	0.03	0.874
**Least-square means**				**Least-square means**			
Region	*est*	*SE*		*Region*	*est*	*SE*	
ACG	-1.78	0.54		ACG	-3.09	0.31	
ANG	-1.94	0.58		ANG	-3.45	0.33	
MFC	-2.68	0.59		MFC	-3.81	0.34	
OFC	-2.83	0.54		OFC	-2.99	0.31	
STG	-3.48	0.58		STG	-3.81	0.33	

Legend: %AO = percentage area occupied by pathology; ACG = anterior cingulate gyrus; ANG = angular gyrus; est = estimate; F = f-value; FTLD-TDP = frontotemporal lobar degeneration with inclusions of the TDP-43 protein; MFC = mid-frontal cortex; OFC = orbitofrontal cortex; SE = standard error; STG = superior temporal gyrus; Type A/Type B/Type C/Type E = subtypes of FTLD-TDP proteinopathy; WM = white matter.

Supplementary Table 14. Regional distribution of GM and WM pathology burden in naPPA with FTLD-Tau and svPPA with FTLD-TDP

**naPPA with FTLD-Tau GM**				**svPPA with FTLD-TDP GM**			
Dependent: ln GM %AO				*Dependent: ln GM %AO*			
Fixed effects:	*df*	*F*	*p*	*Fixed effects:*	*df*	*F*	*p*
**Region**	**4,41**	**8.0**	**0.000**	**Region**	**4,33**	**9.6**	**0.000**
Hemisphere	1,44	4.0	0.052	Hemisphere	1,29	0.0	0.959
Disease duration	1,8	0.2	0.707	Disease duration	1,5	0.5	0.527
**Least-square means**				**Least-square means**			
Region	*est*	*SE*		*Region*	*est*	*SE*	
ACG	-0.01	0.65		ACG	-1.53	0.21	
ANG	-0.75	0.66		ANG	-2.45	0.21	
MFC	0.05	0.64		MFC	-2.54	0.19	
OFC	-1.71	0.65		OFC	-1.34	0.19	
STG	-0.96	0.66		STG	-1.71	0.21	
**naPPA with FTLD-Tau WM**				**svPPA with FTLD-TDP WM**			
Dependent: ln WM %AO				*Dependent: ln WM %AO*			
Fixed effects:	*df*	*F*	*p*	*Fixed effects:*	*df*	*F*	*p*
**Region**	**4,40**	**3.1**	**0.024**	**Region**	**4,36**	**1.4**	**0.267**
Hemisphere	1,43	1.1	0.298	Hemisphere	1,34	0.3	0.564
Disease duration	1,8	0.0	0.853	Disease duration	1,6	5.7	0.057
**Least-square means**				**Least-square means**			
Region	*est*	*SE*		*Region*	*est*	*SE*	
ACG	-0.56	0.68		ACG	-7.47	0.70	
ANG	-0.85	0.70		ANG	-7.28	0.70	
MFC	-0.13	0.67		MFC	-5.97	0.69	
OFC	-1.26	0.68		OFC	-7.00	0.66	
STG	-0.98	0.69		STG	-6.05	0.74	

Legend: %AO = percentage area occupied by pathology; ACG = anterior cingulate gyrus; ANG = angular gyrus; est = estimate; F = F-value; FTLD-Tau = frontotemporal lobar degeneration with inclusions of the tau protein; FTLD-TDP = frontotemporal lobar degeneration with inclusions of the TDP-43 protein; GM = grey matter; MFC = mid-frontal cortex; PPA = nonfluent variant of primary progressive aphasia; OFC = orbitofrontal cortex; p = p-value; SE = standard error; STG = superior temporal gyrus; svPPA = semantic variant of primary progressive aphasia; WM = white matter.

Supplementary Table 15. Regional distribution of GM and WM pathology burden in bvFTD with FTLD-Tau and bvFTD with FTLD-TDP

**bvFTD with FTLD-Tau GM**				**bvFTD with FTLD-TDP GM**			
Dependent: ln GM %AO				*Dependent: ln GM %AO*			
Fixed effects:	*df*	*F*	*p*	*Fixed effects:*	*df*	*F*	*p*
**Region**	**4,91**	**3.3**	**0.015**	**Region**	**4,178**	**5.3**	**0.000**
Hemisphere	1,106	1.5	0.222	Hemisphere	1,185	1.9	0.173
Disease duration	1,20	5.8	0.026	Disease duration	1,40	0.9	0.357
**Least-square means**				**Least-square means**			
Region	*est*	*SE*		*Region*	*est*	*SE*	
ACG	0.82	0.37		ACG	-1.65	0.20	
ANG	-0.43	0.37		ANG	-2.39	0.21	
MFC	0.13	0.38		MFC	-2.18	0.21	
OFC	0.03	0.37		OFC	-1.57	0.19	
STG	-0.35	0.38		STG	-2.13	0.21	
**bvFTD with FTLD-Tau WM**				**bvFTD with FTLD-TDP WM**			
Dependent: ln WM %AO				*Dependent: ln WM %AO*			
Fixed effects:	*df*	*F*	*p*	*Fixed effects:*	*df*	*F*	*p*
**Region**	**4,94**	**2.0**	**0.103**	**Region**	**4,175**	**0.6**	**0.631**
Hemisphere	1,115	2.7	0.102	Hemisphere	1,214	0.2	0.688
Disease duration	1,19	0.3	0.587	Disease duration	1,39	1.2	0.291
**Least-square means**				**Least-square means**			
Region	*est*	*SE*		*Region*	*est*	*SE*	
ACG	-0.71	0.40		ACG	-4.10	0.26	
ANG	-1.57	0.42		ANG	-4.09	0.26	
MFC	-0.61	0.42		MFC	-3.96	0.27	
OFC	-0.95	0.40		OFC	-3.87	0.25	
STG	-0.65	0.42		STG	-3.85	0.26	

Legend: %AO = percentage area occupied by pathology; ACG = anterior cingulate gyrus; ANG = angular gyrus; bvFTD = behavioral variant of frontotemporal dementia; est = estimate; F = F-value; FTLD-Tau = frontotemporal lobar degeneration with inclusions of the tau protein; FTLD-TDP = frontotemporal lobar degeneration with inclusions of the TDP-43 protein; GM = grey matter; MFC = mid-frontal cortex; OFC = orbitofrontal cortex; p = p-value; SE = standard error; STG = superior temporal gyrus; WM = white matter.

Supplementary Table 16. Left-hemisphere sub-analysis: regional distribution of GM and WM pathology burden in naPPA with FTLD-Tau and svPPA with FTLD-TDP in the left hemisphere

**naPPA with FTLD-Tau GM**				**svPPA with FTLD-TDP GM**			
Dependent: ln GM %AO				*Dependent: ln GM %AO*			
Fixed effects:	*df*	*F*	*p*	*Fixed effects:*	*df*	*F*	*p*
**Region**	**4,27**	**7.4**	**0.000**	**Region**	**4,15**	**4.1**	**0.019**
Disease duration	1,6	2.6	0.157	Disease duration	1,15	0.3	0.622
**Least-square means**				**Least-square means**			
Region	*est*	*SE*		*Region*	*est*	*SE*	
ACG	0.07	0.49		ACG	-1.76	0.38	
ANG	-0.93	0.49		ANG	-2.47	0.32	
MFC	0.23	0.47		MFC	-2.68	0.29	
OFC	-1.64	0.49		OFC	-1.30	0.29	
STG	-1.23	0.50		STG	-1.68	0.32	
**naPPA with FTLD-Tau WM**				**svPPA with FTLD-TDP WM**			
Dependent: ln WM %AO				*Dependent: ln WM %AO*			
Fixed effects:	*df*	*F*	*p*	*Fixed effects:*	*df*	*F*	*p*
**Region**	**4,26**	**2.0**	**0.124**	**Region**	**4,14**	**0.9**	**0.471**
Disease duration	1,6	0.7	0.421	Disease duration	1,7	6.2	0.042
**Least-square means**				**Least-square means**			
Region	*est*	*SE*		*Region*	*est*	*SE*	
ACG	-0.72	0.64		ACG	-7.94	0.99	
ANG	-0.79	0.65		ANG	-6.96	0.99	
MFC	-0.28	0.63		MFC	-6.75	0.88	
OFC	-1.51	0.64		OFC	-6.29	0.81	
STG	-0.97	0.65		STG	-5.68	0.99	

Legend: %AO = percentage area occupied by pathology; ACG = anterior cingulate gyrus; ANG = angular gyrus; est = estimate; F = F-value; FTLD-TDP = frontotemporal lobar degeneration with inclusions of the TDP-43 protein; GM = grey matter; MFC = mid-frontal cortex; naPPA = nonfluent variant of primary progressive aphasia; OFC = orbitofrontal cortex; p = p-value; SE = standard error; STG = superior temporal gyrus; svPPA = semantic variant of primary progressive aphasia; WM = white matter.

Supplementary Table 17. Sub-analysis: regional distribution of GM and WM pathology burden in bvFTD with FTLD-Tau and bvFTD with FTLD-TDP in the subset with extended regions

**bvFTD with FTLD-Tau GM**				**bvFTD with FTLD-TDP GM**			
Dependent: ln GM %AO				*Dependent: ln GM %AO*			
Fixed effects:	*df*	*F*	*p*	*Fixed effects:*	*df*	*F*	*p*
**Region**	**2,29**	**5.07**	**0.013**	**Region**	**2,39**	**5.18**	**0.010**
Hemisphere	1,28	0.07	0.795	Hemisphere	1,39	0.40	0.530
Disease duration	1,6	0.40	0.552	Disease duration	1,7	0.37	0.561
**Least-square means**				**Least-square means**			
Region	*est*	*SE*		*Region*	*est*	*SE*	
ACG	1.63	0.66		INS	-1.90	0.43	
INS	1.23	0.64		OFC	-1.89	0.42	
SPL	-0.75	0.58		SPL	-2.76	0.42	
**Post-hoc comparisons**				**Post-hoc comparisons**			
Dependent: ln GM %AO				*Dependent: ln GM %AO*			
Pairwise contrasts	*est*	*SE*	*p*	*Pairwise contrasts*	*est*	*SE*	*p*
ACG - INS	0.40	0.87	0.888	INS - OFC	-0.01	0.32	0.999
ACG - SPL	2.38	0.83	0.021	INS - SPL	0.86	0.32	0.026
INS - SPL	1.98	0.80	0.051	OFC - SPL	0.87	0.31	0.021
**bvFTD with FTLD-Tau WM**				**bvFTD with FTLD-TDP WM**			
Dependent: ln WM %AO				*Dependent: ln WM %AO*			
Fixed effects:	*df*	*F*	*p*	*Fixed effects:*	*df*	*F*	*p*
**Region**	**2,27**	**4.69**	**0.018**	**Region**	**2,40**	**9.45**	**0.000**
Hemisphere	1,26	2.94	0.098	Hemisphere	1,40	0.05	0.824
Disease duration	1,6	0.65	0.450	Disease duration	1,7	0.18	0.681
**Least-square means**				**Least-square means**			
Region	*est*	*SE*		*Region*	*est*	*SE*	
ACG	-0.57	0.80		INS	-3.44	0.39	
INS	0.03	0.83		OFC	-3.37	0.39	
SPL	-2.14	0.78		SPL	-4.46	0.38	
**Post-hoc comparisons**				**Post-hoc comparisons**			
Dependent: ln WM %AO				*Dependent: ln WM %AO*			
Pairwise contrasts	*est*	*SE*	*p*	*Pairwise contrasts*	*est*	*SE*	*p*
ACG - INS	-0.61	0.81	0.737	INS - OFC	-0.07	0.29	0.965
ACG - SPL	1.57	0.74	0.101	INS - SPL	1.02	0.28	0.002
INS - SPL	2.18	0.76	0.021	OFC - SPL	1.09	0.28	0.001

Legend: %AO = percentage area occupied by pathology; ACG = anterior cingulate gyrus; bvFTD = behavioral variant of frontotemporal dementia; est = estimate; F = F-value; FTLD-Tau = frontotemporal lobar degeneration with inclusions of the tau protein; FTLD-TDP = frontotemporal lobar degeneration with inclusions of the TDP-43 protein; GM = grey matter; INS = anterior insular cortex; OFC = orbitofrontal cortex; SE = standard error; SPL = superior parietal lobule; WM = white matter.
